# Amplicon Remodeling and Genomic Mutations Drive Population Dynamics after Segmental Amplification

**DOI:** 10.1093/molbev/msab289

**Published:** 2021-09-28

**Authors:** Andrew B Morgenthaler, Ryan K Fritts, Shelley D Copley

**Affiliations:** Department of Molecular, Cellular and Developmental Biology and Cooperative Institute for Research in Environmental Sciences, University of Colorado Boulder, Boulder, CO, USA

**Keywords:** gene duplication and divergence, amplicon remodeling, molecular evolution, ProA

## Abstract

New enzymes often evolve by duplication and divergence of genes encoding enzymes with promiscuous activities that have become important in the face of environmental opportunities or challenges. Amplifications that increase the copy number of the gene under selection commonly amplify many surrounding genes. Extra copies of these coamplified genes must be removed, either during or after evolution of a new enzyme. Here we report that amplicon remodeling can begin even before mutations occur in the gene under selection. Amplicon remodeling and mutations elsewhere in the genome that indirectly increase fitness result in complex population dynamics, leading to emergence of clones that have improved fitness by different mechanisms. In this work, one of the two most successful clones had undergone two episodes of amplicon remodeling, leaving only four coamplified genes surrounding the gene under selection. Amplicon remodeling in the other clone resulted in removal of 111 genes from the genome, an acceptable solution under these selection conditions, but one that would certainly impair fitness under other environmental conditions.

## Introduction

**Fig. 1.**
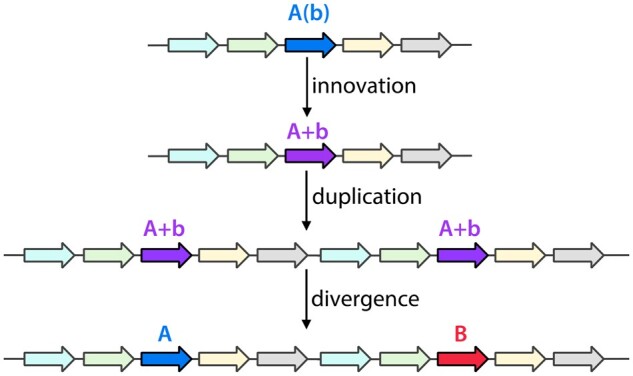
The IAD model for evolution of new genes. “b” indicates an inefficient function of a protein whose primary function is “A.”

Vast superfamilies of enzymes, transcriptional regulators, transporters, and signaling molecules have evolved by gene duplication and divergence. The Innovation-Amplification-Divergence (IAD) model (Bergthorsson et al. 2007) (fig. 1) posits that the process begins when an inefficient secondary function of a protein becomes important for fitness. Gene duplication can improve fitness by increasing gene dosage. The initial event usually duplicates not just the gene under selection, but a surrounding region containing tens or even hundreds of neighboring genes. After the initial duplication, homologous recombination between the extensive regions of homology in the duplicated region can result in rapid amplification. Copy number increases until a balance is reached between the benefit of increasing the level of the inefficient protein and the cost of producing excessive levels of proteins from neighboring genes.

After segmental amplification, mutations in different alleles of the gene under selection allow exploration of sequence space and selection for clones that have improved the newly needed function. When a sufficiently good new protein evolves, the amplified array will shrink to two copies, each containing a specialist gene. However, the process is not finished at this stage. Extra copies of coamplified neighboring genes must still be removed. And the observation that most paralogs are found in distant parts of the genome (Achaz et al. 2001; Katju and Lynch 2003; Bu and Katju 2015) suggests that the final step is often relocation of one of the paralogs. Separating new paralogs may prevent homologous recombination between paralogs that could lead to chimeric sequences or even loss of one gene. The remodeling of the genome during or after successful divergence of a new gene is a critical but understudied part of the IAD process.

**Fig. 2.**
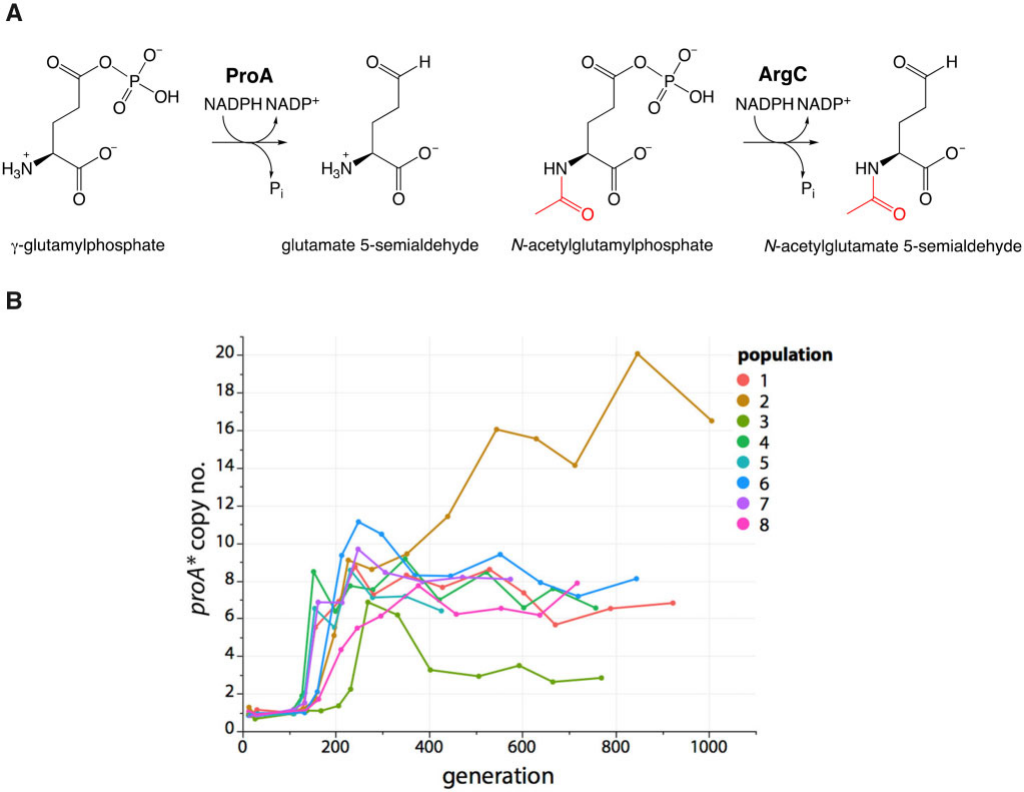
The inefficiency of a promiscuous activity of E383A ProA (ProA*) results in selection for increased copy number of proA* in ΔargC proA* E. coli (AM187) during growth on M9/glucose (0.2%) containing 0.4 mM proline. (*A*) The reactions catalyzed by ArgC and ProA. The substrates for ProA and ArgC differ only by the presence of the acetyl group (red) on *N*-acetylglutamyl phosphate. (*B*) proA* copy number increased in eight replicate populations. Fig. 2*B* reprinted under the Creative Commons Attribution License from Morgenthaler et al. (2019).

We are investigating the IAD process in a strain of *Escherichia coli* that lacks ArgC (*N*-acetyl glutamyl phosphate reductase) (fig. 2*A*), which is essential for synthesis of arginine. We previously discovered that a change of Glu383 to Ala in the active site of ProA (γ-glutamyl phosphate reductase) enables the enzyme (designated ProA* hereafter) to serve the function of ArgC in addition to its native function. However, both activities are inefficient (Khanal et al. 2015), providing selective pressure for gene amplification. We evolved eight populations of a ΔargC proA* strain (AM187) in glucose + proline for 1000 generations to identify mutations that improve synthesis of arginine (Morgenthaler et al. 2019). proA* copy number increased within about 200 generations (fig. 2*B*). In population 3, a mutation in proA* increased the efficiency of the ArgC reaction and resulted in a decrease in proA* copy number from seven to three. None of the other populations acquired a mutation in proA*, although growth rate increased 2.5–3.5-fold as a result of mutations elsewhere in the genome.

**Fig. 3.**
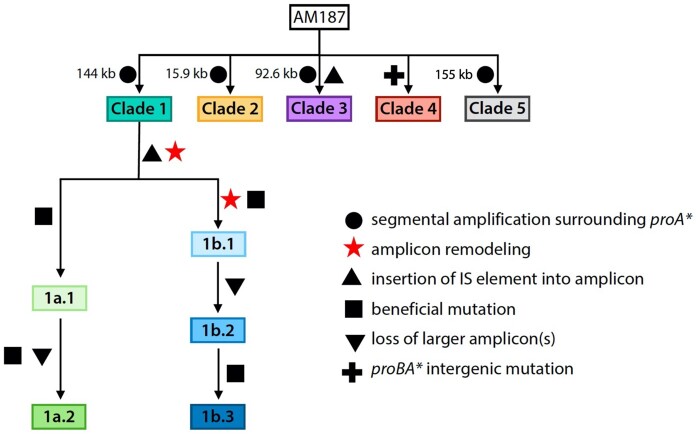
Summary of genetic changes in five dominant clades during the 1006-generation evolution of strain AM187. Clades 2–5 were lost by generation 669.

A striking feature of the data in figure 2*B* is the fluctuation in proA* copy number during the experiment. We have identified the cause of the fluctuations in population 2, in which the highest copy number and most extreme fluctuations occurred. Dynamics within the evolving population were driven by multiple factors, including the size of the amplified region surrounding proA*, beneficial mutations elsewhere in the genome, and remodeling within amplicons that reduced the number of coamplified genes. Figure 3 summarizes the genetic changes in population 2 over the course of 1006 generations in five dominant clades detected at generation 227. By generation 1006, the population was dominated by descendants of Clade 1 that had minimized amplicon size by one or two episodes of amplicon remodeling. These data show that the process of removal of extraneous neighboring genes can begin long before divergence results in a new protein.

## Results

### A Large-Scale Genomic Rearrangement Had Occurred in the Parental Strain AM187

Population 2 was evolved from the previously created ΔargC proA* E. coli strain AM187 (Morgenthaler et al. 2019). One of the two promoter mutations that occurred during previous evolution of ΔargC proA* E. coli on glucose (Kershner et al. 2016) had been introduced into strain AM187 to ensure homogeneity in the promoter during the evolution experiment. In addition, *fimAICDFGH* and *csgBAC* had been deleted to minimize formation of biofilms, and *yfp* had been inserted following the proBA* operon. We had previously sequenced the AM187 genome using short-read sequencing. In this work, we resequenced the genome using long-read sequencing. The long-read sequencing indicated that a 1.8-Mb inversion in the genome had occurred between two IS3 elements (supplementary fig. 1, Supplementary Material online). The corrected genome has been uploaded to GenBank (accession number CP037857.2).

### Population Genome Sequencing Reveals Amplifications and Mutations in the Evolving Population

**Fig. 4.**
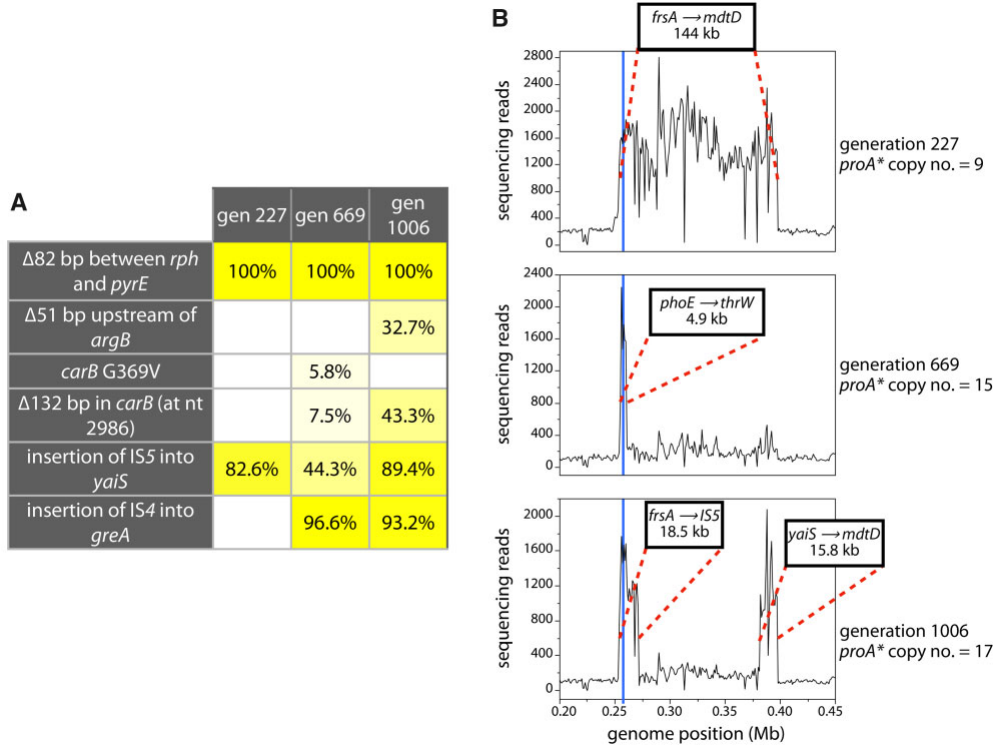
Prevalent mutations and amplifications observed in population 2 at generations 227, 669, and 1006. (*A*) Frequencies of relevant mutations that influenced clonal dynamics. (*B*) Sequencing reads mapped onto the genome of AM187, showing changes in the region of amplification during the experiment. The blue line indicates the position of the proBA* operon. ProB (γ-glutamyl kinase) synthesizes the substrate for ProA.

We sequenced population genomic DNA from population 2 at generations 227, 669, and 1006. The most relevant mutations detected at a frequency of > 5% are shown in figure 4*A*. (Mutations detected at each timepoint are listed in supplementary table 1, Supplementary Material online.) The importance of each of these mutations will be discussed below. The region of amplification in the population as a whole changed throughout the experiment (fig. 4*B*). At generation 227, a 144-kb region was amplified 9-fold. By generation 669, a much smaller 4.9 kb region surrounding proA* was amplified by 15-fold; the original 144 kb region was still amplified, but its copy number had decreased dramatically. The picture at generation 1006 is complex. The 4.9-kb amplicon is still evident, but a larger 18.5-kb region and a downstream 15.8-kb region are also amplified. These data are consistent with competition between clones with different size amplicons, amplicon remodeling, or both.

### Five Clades Dominated the Population by Generation 227

By generation 227, an 82-bp deletion between *rph* and *pyrE* had nearly swept the population. This deletion, which relieves a pyrimidine synthesis deficiency in K12 strains of *E. coli*, is commonly found during adaptive evolution of K12 strains (Bonekamp et al. 1984; Jensen 1993; Conrad et al. 2009; Blank et al. 2014; Knoppel et al. 2018) and occurred in all eight of our previously evolved populations (Morgenthaler et al. 2019). We also detected a C to A mutation 55 bp upstream of *pyrE* in a colony isolated from the population at generation 227. Several point mutations in this region have been reported to occur during evolution of K12 strains on glucose (Blank et al. 2014; Knoppel et al. 2018), so this mutation likely improves pyrimidine synthesis, as well.

We interrogated the genotypes of 37–40 colonies isolated at generations 227, 669, and 1006 using a suite of polymerase chain reaction (PCR) primers (supplementary fig. 2 and supplementary table 2, Supplementary Material online) and/or short-read whole-genome sequencing. The primers were designed to detect the amplicon boundaries, deletions, and IS element insertions that were detected in population genomic DNA at each timepoint. The locations of primers used to amplify fragments spanning amplicon boundaries were suggested by the amplified regions visible in population genomic DNA (fig. 4). Junction sequences were determined by sequencing these PCR amplicons for Clades 1–4 and by short-read whole-genome sequencing for Clade 5. These data are summarized in supplementary figures 3–6 and supplementary tables 3–5, Supplementary Material online.

**Fig. 5.**
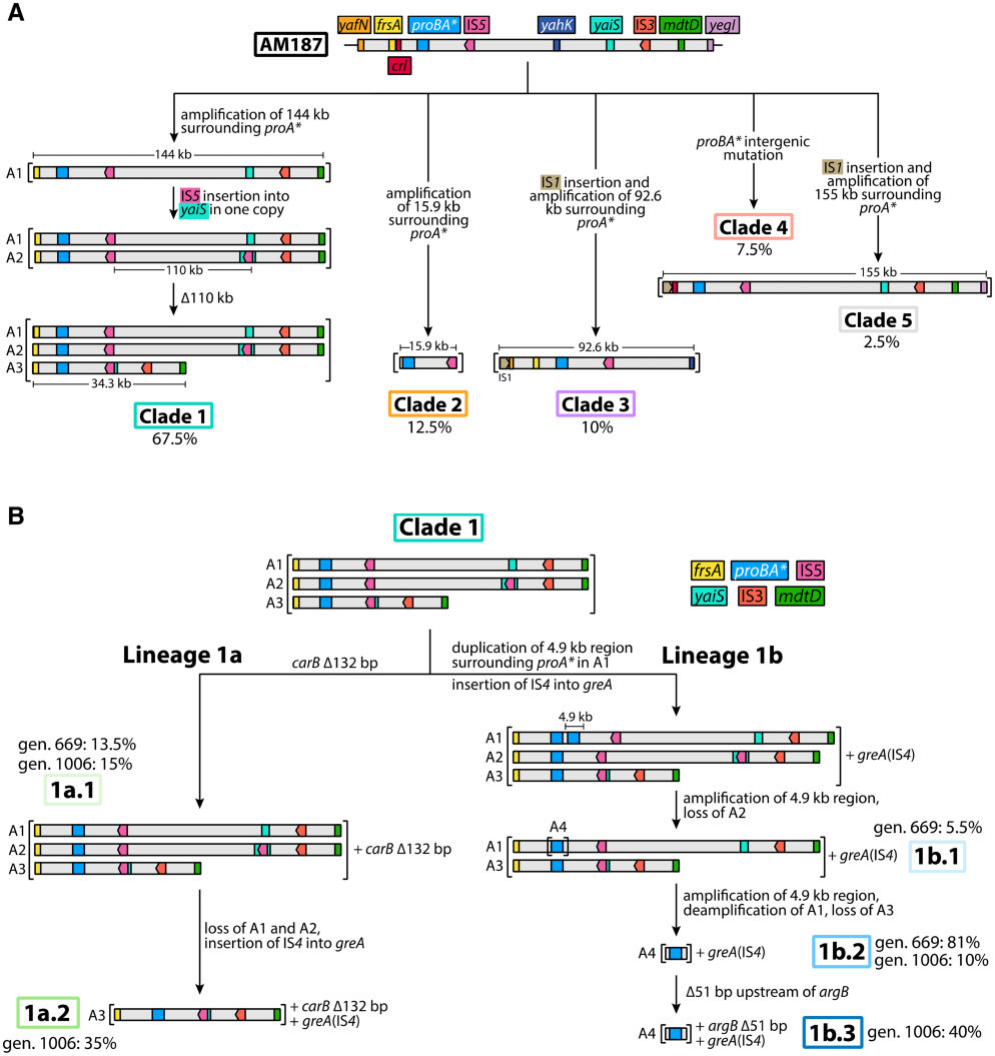
Genetic changes that occurred during the evolution of population 2 over 1006 generations. (*A*) Genotypes of five clades detected by generation 227. (*B*) Genotypes of lineages derived from Clade 1 after generation 227. Brackets indicate amplicons. The diagrams are not to scale.

At generation 227, the population contained five dominant clades. Clades 1, 2, 3, and 5 initially amplified regions of 144, 15.9, 92.6, and 155 kb, respectively (figs. 3 and 5). Clade 4 had acquired a G to A mutation at -4 relative to the start codon of proA*. We previously found that a point mutation at -3 relative to the start codon of proA* in ΔargC proA* Salmonella enterica increases expression of ProA* by 2.7-fold (Kristofich et al. 2018), likely due to decreased secondary structure upstream of the start codon and consequently greater accessibility to the ribosome. To test whether the point mutation in Clade 4 increases translation of proA* mRNA, we fused a segment encompassing 150 bp upstream of the proBA* operon through the first 171 bp of proA* to *gfp* in plasmid pACYC177. (Plasmids used in this work are described in supplementary table 6, Supplementary Material online.) The mutation at -4 increases translation efficiency by 1.8-fold (supplementary fig. 7, Supplementary Material online).

Clade 1 was the most abundant at generation 227, despite carrying a large 144-kb amplicon. Clades 2 and 3, which have smaller amplicons, should be more fit because they can sustain more copies of proA* and can therefore provide more of the weak-link enzyme ProA*. Thus, we suspect that Clade 1 arose first in the population. This supposition is consistent with the observation that the Clade 1 junction indicates that recombination occurred within a 23-bp region with only six mismatches. This event would likely have occurred more readily than those that generated the junctions in Clades 2, 3, and 5, which occurred in regions with little sequence identity (supplementary table 7, Supplementary Material online). An alternative, although perhaps less likely, explanation is that one or more genes that were coamplified in the Clade 1 amplicon but not in the smaller Clade 2 and 3 amplicons provided a fitness benefit under these growth conditions.

### Amplicon Remodeling Occurred before Generation 227 in Clade 1

By generation 227, Clade 1 had already undergone amplicon remodeling. This remodeling was facilitated by insertion of IS*5* into *yaiS*, which encodes a putative deacetylase, in a copy of the initial 144-kb amplicon (A1) to form amplicon A2 (fig. 5*A*). This insertion was identified in 68% of the 40 colonies we analyzed (supplementary table 3, Supplementary Material online). An IS*5* element was already present in A1. Deletion of 110 kb of genomic DNA between the two IS*5* elements in A2 generated a much smaller amplicon (A3) (fig. 5*A*). Notably, all of the 27 Clade 1 colonies we analyzed contained all three types of amplicons. A1 is indicated by the presence of wild-type *yaiS*, and A2 by the insertion of IS*5* in *yaiS* (supplementary fig. 4*a*, Supplementary Material online). A3 is indicated by a 110-kb deletion between the two IS*5* elements in A2 (supplementary fig. 4*b*, Supplementary Material online). (The 110-kb deletion includes 109 kb of genomic DNA and 1 kb of IS*5*.)

### Clade 1 Out-Competed Clades 2 and 3 due to Acquisition of Beneficial Mutations and Additional Amplicon Remodeling

Clades 2, 3, 4, and 5—which comprised 33% of the population at generation 227—were undetectable in population genomic DNA by generation 669. Two lineages derived from Clade 1 were identified among 37 colonies at generation 669. These lineages had acquired beneficial mutations elsewhere in the genome that allowed them to outcompete the other clades.

Lineage 1b dominated the population at generation 669. An amplification of a 4.9-kb region surrounding the proBA* operon was observed in this lineage at generation 669; the previously amplified 144-kb region was still present in the population, but at a lower level (fig. 4). This small amplicon (A4) arose within a copy of the A1 amplicon. We know that it did not arise in A2 or A3 because the PCR signatures of those amplicons—the insertion of IS*5* into *yaiS* for A2 and the 110-kb deletion between IS*5* elements for A3—were lost by generation 1006 whereas the 4.9-kb amplicon remained (supplementary figs. 6*b* and *c*, Supplementary Material online). We captured intermediates in this process in two lineage 1b.1 colonies. One colony (C35) retained A3, but showed only a faint band with primers diagnostic for A2, whereas another (C36) showed only a faint band with primers diagnostic for A3 and had completely lost A2 (supplementary figs. 5*b* and *c*, Supplementary Material online). The lineage 1b.2 colonies had all lost both A2 and A3 by generation 669. Importantly, the original amplification junction was no longer present, indicating that the A1 amplicon in which A4 arose had deamplified to a single copy (supplementary fig. 5*a*, Supplementary Material online). The loss of the large amplicons enabled by amplification of A4 returned the copy numbers of 150 genes to normal. Within the A4 amplicon, only four genes surrounding the proBA* operon were coamplified.

Lineage 1b had also acquired an insertion of IS*4* into *greA* by generation 669. GreA is a transcription factor that rescues backtracked and stalled transcription elongation complexes by accelerating hydrolysis of extruded RNA blocking the substrate binding site (Sosunova et al. 2003). Insertion of IS*4* into *greA* had previously been observed during evolution of *E. coli* K12 in glucose (Knoppel et al. 2018), and also appeared in lineage 1a by generation 1006 (see further below). The recurrence of this mutation in two parallel lineages suggests that it is beneficial. We inserted IS*4* into *greA* in AM187 but found that it did not increase growth rate (supplementary fig. 8, Supplementary Material online). Thus, loss of GreA activity may be beneficial only in the genetic background in which it occurred, that is, in the context of large segmental amplifications surrounding proA*. A possible explanation is suggested by a report that deletion of *greA* stimulates RecA-dependent recombination in *E. coli*, possibly by increasing collisions between replication forks and stalled transcription elongation complexes that generate recombinogenic double-stranded breaks (Poteete 2011)*.* The insertion of IS*4* into *greA* in lineage 1b may have provided a competitive advantage due to accelerated amplification of the small amplicon A4 and loss of the large amplicons A2 and A3, which would occur via RecA-mediated unequal crossing over events.

Lineage 1a.1, which accounted for 13.5% of the colonies at generation 669, had acquired a 132-bp deletion in *carB* (supplementary fig. 5*d*, Supplementary Material online). CarB is the beta subunit of carbamoyl phosphate synthetase, which provides carbamoyl phosphate for synthesis of both arginine and pyrimidines. We previously showed that this deletion increases growth rate of the parental AM187 strain by 54% (Morgenthaler et al. 2019). The deletion has little effect on catalytic activity, but abolishes feedback inhibition of carbamoyl phosphate synthetase by UMP, thus increasing the amount of carbamoyl phosphate available for arginine synthesis.

Amplicons A1, A2, and A3 were still present in most of the 1a.1 colonies at generation 669. Interestingly, one colony (C10, supplementary fig. 5*b*, Supplementary Material online) had lost A1, the first indication of selective loss of a larger amplicon in this lineage. By generation 1006, lineage 1a.2 colonies had lost both A1 and A2, but still retained A3 (supplementary fig. 6*a*–*c*, Supplementary Material online). They had also acquired the insertion of IS*4* into *greA* that occurred earlier in lineage 1b (supplementary fig. 6*f*, Supplementary Material online).

Between generations 669 and 1006, lineage 1b acquired an additional beneficial mutation, a deletion of 51 bp upstream of *argB* (supplementary fig. 6*g*, Supplementary Material online). We previously showed that mutations upstream of *argB* increase the level of ArgB by 3–6-fold by increasing translation of the *argB* mRNA (Morgenthaler et al. 2019). ArgB synthesizes *N*-acetyl glutamyl phosphate, the substrate for ArgC in wild-type cells and for the weak-link ProA* in the ΔargC proA* cells. Overproduction of ArgB would be expected to result in a higher level of *N*-acetyl glutamyl phosphate, which should push material through the bottleneck in the arginine synthesis pathway.

## Discussion

The IAD model describes a convincing mechanism for the evolution of a new function by gene duplication/amplification and divergence. However, it does not address the genomic remodeling that must take place to remove extra copies of coamplified genes. Genomic remodeling clearly occurs, but how and when has received little attention. The Roth group identified amplicon remodeling in *S. enterica* carrying a leaky frameshift mutation in the *lacI* portion of a *lacI-lacZ* fusion on an F′_128_ plasmid. These cells produce only 2% of the wild-type level of β-galactosidase (Kugelberg et al. 2006, 2010) and thus cannot grow on lactose minimal medium unless a pre-existing duplication encompassing *lac* enables further amplification. *lac* is particularly prone to duplication on the F′_128_ plasmid due to the presence of two IS*3* elements that facilitate a 134-kb duplication and REP elements that facilitate smaller duplications (18–49 kb). Further, the DNA ends generated by the transfer machinery are believed to stimulate duplication and amplification. The combination of these factors results in duplication of *lac* in 0.2% of the cells even under nonselective conditions (Kugelberg et al. 2006). Cells containing the large 134 kb duplication can generate 10 copies of *lac*, but grow slowly and do not form visible colonies on lactose minimal medium. Some of these cells, primarily those in which the gene encoding the error-prone polymerase DinB is coamplified (Slechta et al. 2003), acquire a mutation that reverts the *lac* frameshift, leading to rapid growth and loss of unmutated amplicons. In others, deletion within an amplicon allows accumulation of as many as 100 copies of the leaky *lac* allele, enough to support rapid growth. This experimental system requires that *lac* be encoded on a conjugative plasmid (Galitski and Roth 1995). (The parental cells are unable to grow on lactose, and thus cannot replicate their DNA, a prerequisite for chromosomal gene duplication. However, the plasmid DNA can be replicated even in non-growing cells (Galitski and Roth 1995), providing an opportunity for amplification of *lac* to a level that can support growth.) Our work extends the foundational work of the Roth group (Kugelberg et al. 2006, 2010) by 1) placing a gene under selection in its normal genomic context; 2) carrying out experiments in liquid medium, conditions under which clones compete with one another; 3) following remodeling over a longer period of time (1,000 generations vs. the approximately 30 generations it takes to produce 10^9^ cells in a colony); and 4) considering the impact of mutations elsewhere in the genome as well as amplicon structure on clonal dynamics in an evolving population.

Amplification of different genomic regions surrounding the proBA* operon occurred early in our evolution experiments. Thirty-seven of the 40 colonies we interrogated at generation 227 already had an amplified region, attesting to the strong fitness benefit provided by an increase in proA* copy number. The Clade 1 junction occurred in a 23-bp region with only five mismatches. However, the junction sequences in Clades 2, 3, and 5 show little sequence identity between the sequences that are fused (supplementary table 7, Supplementary Material online). Similarly unimpressive junction sequences formed between regions of low sequence identity have been observed in previous studies (Brochet et al. 2008; Seaton et al. 2012) and may arise by multiple mechanisms (Reams and Roth 2015). Regardless of the mechanism by which they formed, the amplicons we observed are large, and their size imposes a significant fitness burden that is alleviated in Clade 1 by remodeling within a few hundred generations.

In this work, we observed two different mechanisms for amplicon remodeling. In lineage 1a, a deletion promoted by insertion of IS*5* into amplicon A1 removed 109 kb of genomic DNA (110 kb total including the loss of IS*5* sequences), a total of 111 genes, leaving behind a smaller 34 kb amplicon (A3). In lineage 1b, a small 4.9kb region amplified within a copy of the large A1 amplicon. As this region (A4) amplified, other copies of A1 as well as A2 and A3 were gradually lost, leaving a single copy of A1 and multiple copies of A4, which contains only four genes in addition to the proBA* operon. We observed heterogeneity in amplicons resulting from remodeling within individual colonies. At generation 227, Clade 1 cells contained three different amplicons (A1, A2, and A3). At generation 669, one lineage 1b colony carried A1 and A3 as well as the new amplicon A4 within one copy of A1. Notably, if we had not sampled the population at early time points, we would have missed the complicated histories of both A3 and A4.

This work, as well as the previous study by Kugelberg et al. (2006), demonstrates that extra copies of coamplified genes can be removed by amplicon remodeling even before the gene under selection begins to acquire mutations. This finding is perhaps not surprising. Segmental amplifications and deletions are orders of magnitude more common than point mutations (Lynch et al. 2008; Lipinski et al. 2011; Schrider et al. 2013), particularly when insertion elements are involved. The insertion of IS*5* into *yaiS* in A1 facilitated a deletion that removed 111 extraneous genes. On the other hand, improvement of an inefficient enzyme activity may require specific mutations. Thus, the mutational target size may be small. This is certainly the case in this experimental system. Over the course of 1000 generations in eight replicate populations, a beneficial mutation in proA* arose only once, in population 3 (Morgenthaler et al. 2019).

Interestingly, the selective pressure to reduce amplicon size led to an outcome in Clade 1 that is certainly undesirable in the long term. By generation 1006, lineage 1a retained only A3. Loss of the larger amplicons resulted in the complete loss of 111 genes from the genome. Most of these were prophage genes or genes of unknown function (supplementary table 7, Supplementary Material online). Clearly none is essential for growth under these conditions. However, genes for degradation of lactose, 3-phenylpropionate and 3-(3-hydroxyphenyl)propionate were deleted, so lineage 1a sacrificed the ability to grow on these carbon sources in order to optimize synthesis of arginine.

Amplicon remodeling and selective loss of large amplicons, however, were not the only drivers of clonal dynamics in the evolving population. We identified three beneficial mutations that contributed to fitness and clonal expansion at different times during the experiment. We previously showed that mutations upstream of *argB* and in *carB* improve arginine synthesis in Δ*argC E. coli* by either pushing or pulling, respectively, material through the bottleneck in the pathway caused by the inefficiency of ProA* (Morgenthaler et al. 2019). A 58-bp deletion upstream of *argB* in population 6 increases the level of ArgB by 6.3-fold, probably by decreasing secondary structure around the ribosome binding site. When introduced into the parental AM187 strain, this 58-bp deletion increases growth rate by 61%. The 51-bp deletion in lineage 1a encompasses all but 7 bp of this region, so should have a similar effect. Overexpression of ArgB should produce more *N*-acetylglutamyl phosphate and increase the rate at which *N*-acetylglutamyl semialdehyde is produced by ProA*. The 132-bp deletion in *carB* abolishes feedback inhibition of carbamoyl phosphate synthetase by UMP, and when introduced into the parental AM187 strain, increases growth rate by 47%. This mutation should enable the enzyme to produce more carbamoyl phosphate, a cosubstrate for the ornithine transcarbamoylases (ArgF and ArgI) downstream of ProA*, and help pull material through the arginine synthesis pathway.

**Fig. 6.**
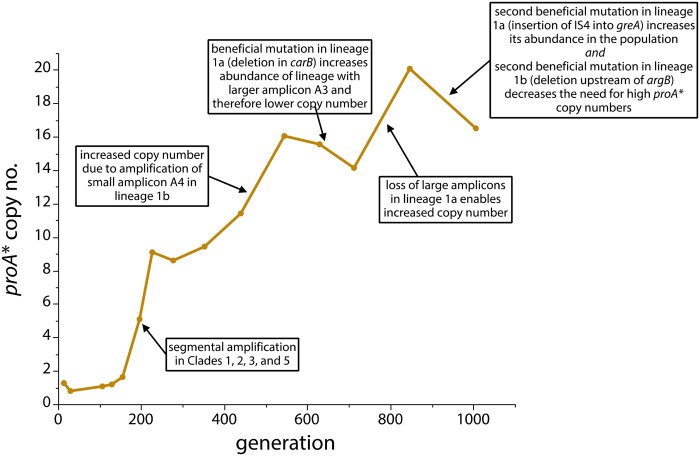
A plausible series of events responsible for fluctuations in proA* copy number during the evolution experiment.

The observation of amplicon remodeling events, selective loss of large amplicons and beneficial mutations elsewhere in the genome allows us to postulate a plausible explanation for the fluctuations in proA* copy number during the evolution of population 2 (fig. 6). The initial increase in copy number is due to the expansion of Clades 1, 2, 3, and 5, each of which amplified a segment of the genome surrounding proBA*. Clades 2 and 3 amplified much smaller segments than Clade 1, and would have been expected to dominate the population; smaller amplicons impose a smaller metabolic burden and thus can accumulate to higher levels and produce more ProA*. (This supposition is supported by the observation that the copy number of proA* increased after the small A4 amplicon emerged [fig. 4]). However, lineage 1a acquired a mutation in *carB* and lineage 1b acquired a mutation in *greA* and a duplication of 4.9 kb surrounding proBA* inside a copy of A1. These mutations allowed Clade 1 to out-compete Clades 2, 3, 4, and 5. The small decrease in copy number after generation 200 is likely due to loss of Clades 2 and 3, which had relatively small amplicons and therefore likely higher proA* copy numbers. The subsequent increase in copy number may have been due to the increasing prevalence of lineage 1b, which acquired higher copy numbers as the 4.9-kb amplicon A4 amplified. The transient decrease in copy number around generation 600 was likely due to a resurgence of lineage 1a, which can maintain fewer copies of proA* because of its large amplicons, when it acquired the highly beneficial mutation in *carB*. Subsequently, amplicon remodeling in lineages 1a and 1b followed by selective loss of large amplicons enabled a further increase in proA* copy number. Finally, copy number dipped again by generation 1006 for two reasons. Lineage 1a, which carried A3, acquired a second beneficial mutation (insertion of IS*4* into *greA*) and expanded to 50% of the population. The large size of A3 limited its copy number to about 11 at this stage. Second, the deletion upstream of *argB* in lineage 1b.2 apparently improved arginine synthesis to the point that the proA* copy number dropped to about 6, a significant decrease from the 13 copies present in lineage 1b at generation 669.

## Conclusion

The role of gene duplication and divergence in the evolution of new protein functions is clear from the evolutionary record. However, we typically view the end result of the process. The results described here reveal the complex dynamics of the early stages as clones rise and fall due to stochastic events that amplify regions of different sizes, remodel amplicons, and mutate genes elsewhere in the genome. Three broadly relevant principles emerge from our results. First, amplicon remodeling happens quickly, driven by the benefit of decreasing protein production from coamplified genes. Second, both amplicon size and mutations elsewhere in the genome contribute to fitness; a strongly beneficial mutation elsewhere in the genome can compensate for the costs of a large amplicon and indeed allow fewer copies to suffice. Third, the entire process is driven by expediency. Any mutation that increases fitness under the selection conditions will be favored, even if it damages a previously well-evolved function. Amplicon remodeling in lineage 1a resulted in a massive loss of 111 genes from the genome, which increased fitness by diminishing the cost of expressing coamplified genes. This solution was tolerable under these selection conditions—growth on glucose + proline—but this clone would be less fit if conditions changed and the lost genes were important for fitness or even survival. If the selection had been carried out in lactose minimal medium, this particular expedient solution would not even have been possible. Expedient mutations also occurred elsewhere in the genome, in *carB* and *greA*, causing loss of a key regulatory function and total loss of function, respectively. In contrast to the loss of 111 genes in lineage 1a, these mutations could potentially be repaired after arginine synthesis was improved by evolution of a neo-ArgC from ProA*. Removal of IS*4* from *greA* should be easily accomplished, but repair of *carB* would require horizontal gene transfer to compensate for the loss of 132 bp of coding sequence. Taken together, our findings suggest that the presence of suitably specific, efficient, and well-regulated paralogs in a genome belies the turbulent processes necessary to achieve that end.

## Materials and Methods

### Materials

Common chemicals were purchased from Sigma-Aldrich (St Louis, MO) and Fisher Scientific (Fair Lawn, NJ). Primers and plasmids used in this study are listed in supplementary tables 2 and 6, Supplementary Material online, respectively.

### Culture Conditions

*E.* *coli* cultures were grown at 37 °C with shaking in LB medium or M9/glucose (0.2%) containing 0.4 mM proline. Kanamycin (20 µg/ml), ampicillin (100 µg/ml) or chloramphenicol (20 µg/ml) was added as required.

### Strain Construction

Construction of the parental strain AM187 was described previously (Morgenthaler et al. 2019). To construct *greA*::IS*4* AM187, cells were first transformed with the temperature-sensitive pSIM6 plasmid encoding λ Red recombinase genes (*exo*, *gam*, and *bet*) under control of a heat-inducible promoter (Datta et al. 2006). Cells were allowed to recover at 30 °C for 2–3 h and then plated on LB/ampicillin. The anhydrotetracycline-inducible selection/counter-selection cassette *tetR*-*ccdB*-*cat* was amplified from pDLM3 (Bossi et al. 2019) and ∼550-bp fragments of sequences flanking *greA* were added by overlap extension PCR to construct the Δg*reA*::*tetR*-*ccdB*-*cat* cassette. Cells harboring pSIM6 were grown to an OD_600_ of 0.3–0.6 at 30 °C and then incubated at 42 °C with shaking for 15 min to induce expression of the λ Red recombinase genes. The cells were then subjected to centrifugation at 4 °C, washed with ice-cold deionized water, and immediately subjected to electroporation with 0.5–1.5 μg of the Δg*reA*::*tetR*-*ccdB*-*cat* cassette. Cells were allowed to recover at 30 °C for 2–3 h before being spread onto LB/chloramphenicol (20 µg/ml) plates. Genomic integration of *tetR*-*ccdB*-*cat* at the *greA* locus in chloramphenicol-resistant colonies was confirmed by colony PCR and Sanger sequencing. The *tetR*-*ccdB*-*cat* cassette was then replaced with *greA*::IS*4* amplified from genomic DNA of an evolved isolate with ∼550-bp regions flanking *greA*. The Δ*greA*::*tetR*-*ccdB*-*cat* strain harboring pSIM6 was subjected to a second round of λ Red-mediated genome editing as described above with electroporation of 0.1–0.5 μg of the *greA*::IS*4* linear fragment. Cells were plated on LB/anhydrotetracycline (4–8 µg/ml) and integration of *greA*::IS*4* in chloramphenicol-sensitive colonies was confirmed by colony PCR and Sanger sequencing. Individual colonies were cured of pSIM6, which encodes a temperature-sensitive replication protein, by overnight growth at 40 °C. Loss of pSIM6 was confirmed by loss of ampicillin resistance.

### Laboratory Evolution

The evolution of eight replicate cultures of AM187 for approximately 1,000 generations in a turbidostat at 37 °C in M9/glucose (0.2%) containing 0.4 mM proline and 20 µg/ml kanamycin was described previously (Morgenthaler et al. 2019). A 3-ml portion of each population was collected every 2–3 days; 800 µl was used to make a 10% glycerol stock, which was stored at −70 °C. The remaining sample was pelleted for purification of genomic DNA using the Invitrogen PureLink Genomic DNA Mini Kit according to the manufacturer’s protocol. The copy number of proA* was determined by qPCR using purified population genomic DNA as described previously (Morgenthaler et al. 2019).

### Whole-Genome Sequencing

Libraries were prepared from genomic DNA from clones or populations using a modified Illumina Nextera protocol (Baym et al. 2015) and sequenced on an Illumina NextSeq500 to produce 151-bp paired-end reads (60–130-fold coverage). Reads were trimmed using fastp v0.20.0 (Chen et al. 2018) and mapped using *breseq* v0.35.5, using the default consensus mode for clones and the polymorphism (mixed population) option for populations (Deatherage and Barrick 2014).

Long-read sequencing of AM187 was performed at the Microbial Genome Sequencing Center (Pittsburgh, PA) using the Oxford Nanopore platform. Reads were trimmed using Porechop v0.2.4 and mapped using minimap2 v2.17 (Li 2018) with the default Oxford Nanopore settings. The resulting SAM file was converted into a sorted and indexed BAM alignment using SAMtools 1.11 (Li et al. 2009).

### Identification of Amplification Junctions

The amplification junction in the single Clade 5 colony at generation 227 was identified by whole-genome sequencing. Amplification junctions in the other clades were initially tested by PCR using the primers listed in supplementary table 2, Supplementary Material online, and diagrammed in supplementary figure 2, Supplementary Material online. Frozen stocks of the populations at generations 227, 669, and 1006 were streaked onto plates containing M9/glucose (0.2%), 0.4 mM proline and 20 µg/ml kanamycin. Forty colonies from each time point were resuspended individually in 20 µl of water. Two µl of the suspended cells were used to inoculate 3 ml of M9/glucose (0.2%) containing 0.4 mM proline and 20 µg/mL kanamycin. The cultures were grown to saturation with shaking at 37 °C and stored at −70 °C as a 10% glycerol stock. One microliter of the remaining cell suspension was used to check for amplification junctions in reaction mixtures containing 0.2 µl of each 10 µM primer, 3.6 µl water, and 5 µl OneTaq Hot Start Quick-Load 2x Master Mix with Standard Buffer (NEB). The manufacturer’s instructions were followed for PCR conditions using annealing temperatures determined based on the NEB Tm calculator (http://tmcalculator.neb.com/#!/main, last accessed September 30, 2021). Purified AM187 genomic DNA was used as a control for each PCR. PCR amplicons were sequenced by Sanger sequencing to identify amplification junctions (supplementary table 7, Supplementary Material online).

### Diagnostic PCRs

Diagnostic PCRs using the primers listed in supplementary table 2, Supplementary Material online, and diagrammed in supplementary figure 2, Supplementary Material online, were carried out to identify the presence or absence of mutations detected by sequencing of population genomic DNA in genomic DNA isolated from individual colonies at each timepoint. PCRs were carried out as described above.

### Determination of the Effect of the Intergenic Mutation Upstream of proA* on Translation Efficiency

The region from 150 bp upstream of proBA* through the first 171 bp (57 codons) of proA* was amplified by PCR from AM187 genomic DNA and from a Clade 4 clone genomic DNA. *gfp* with a T7 terminator sequence after the stop codon was amplified from pGRG36-Kn-PA1-GFP (Yang et al. 2020). These fragments were assembled with the linearized backbone of pACYC177 by Gibson assembly using 20-bp overlap regions. The assembly products (pWTproA57-gfp and pClade4mutproA57-gfp) were transformed into chemically competent *E. coli* DH5α by heat shock at 42 °C for 30 s. Cells were allowed to recover at 37 °C for 1–2 h and then plated on LB/ampicillin. Proper construction of the proBA*-gfp fusions was confirmed by Sanger sequencing. The pWTproA57-GFP and pClade4mutproA57-GFP plasmids (50–100 µg) were introduced into AM187 by electroporation. Cells were allowed to recover at 37 °C for 1–2 h before plating on LB/ampicillin. Successful electroporation was confirmed by colony PCR of fluorescent ampicillin-resistant colonies.

Aliquots (∼20 μl) of thawed frozen stocks of AM187 harboring pClade4mutproA57-GFP or pWTproA57-GFP were inoculated into 4 ml of M9/glucose (0.2%) containing 0.4 mM proline and 20 µg/ml kanamycin (three replicate cultures). Three tubes were inoculated with AM187 in the same medium lacking kanamycin. The cultures were grown overnight at 37 °C with shaking. Each culture (OD_600_ 0.1–0.3) was then diluted 1:4 into fresh medium and grown for 8 h at 37 °C to exponential phase (OD_600_ 0.15–0.4). Two hundred microliters of each culture were transferred to wells of a 96-well plate with black sides and a clear bottom (Corning Costar). GFP fluorescence was measured using a BioTek Synergy H1 microplate reader (excitation 485 nm; emission 515 nm). The fluorescence signal from sterile medium was subtracted from all readings. The OD_600_ of each sample was also measured with the BioTek Synergy H1 microplate reader and used to normalize GFP fluorescence (RFU) to cell density (OD_600_).

### Determination of the Effect of Insertion of IS*4* into *greA*

Specific growth rates for AM187 and *greA*::IS*4* AM187 were calculated from growth curves measured in quadruplicate. Overnight cultures were grown in LB/kanamycin at 37 °C from glycerol stocks. Forty microliters of each overnight culture was used to inoculate 4 ml of LB/kanamycin and the cultures were grown to mid-log phase (OD_600_ 0.3–0.6) at 37 °C with shaking. The cultures were subjected to centrifugation at 4,000 × g for 10 min at room temperature and the pellets resuspended in an equal volume of phosphate-buffered saline (PBS). The pellets were washed once more in PBS. The cells were resuspended to an OD_600_ of 0.001 in M9/glucose (0.2%) containing 0.4 mM proline and 20 µg/ml kanamycin and 100 microliter aliquots were transferred into wells of a 96-well plate. The plates were incubated in a Varioskan plate reader (Thermo Scientific) at 37 °C with shaking for 1 min every 5 min. The OD_600_ was measured every 20 min. The OD_600_ of control wells containing sterile medium was subtracted from each point of the growth curve. Specific growth rates were calculated by fitting the data to the modified Gompertz equation (Zwietering et al. 1990).

## Supplementary Material

Supplementary data are available at *Molecular Biology and Evolution* online.

## Supplementary Material

msab289_Supplementary_Data
