# Supplementary material for: Amplicon Remodeling and Genomic Mutations Drive Population Dynamics after Segmental Amplification
Source: Mol Biol Evol. 2021 Sep 28;39(1):msab289. doi: 10.1093/molbev/msab289 (PMC8763031; doi:10.1093/molbev/msab289)
Supplement: msab289_Supplementary_Data [file msab289_supplementary_data.pdf]

Supplementary Tables and Figures

Table S1. Mutations detected at a frequency of >5% by population genome sequencing of population 2 at generations 227, 669 and 1006. Mutations discussed in the text are highlighted in red. Arrows refer to the direction of the gene in the *E. coli* genome.

Generation 227

position	mutation	Freq (%)	annotation	gene	description
381,062	insertion of IS5 into *yaiS*	82.6	insertion in the antisense direction at 16/558 of *yaiS*, with duplication of nts 13-16 of *yaiS* following the insertion	*yaiS* ←	putative deacetylase
3,808,881	Δ82 bp	100	deletion in *rph* increases expression of downstream gene *pyrE*	*rph* ←	truncated RNase PH

Generation 669

position	mutation	Freq (%)	annotation	gene	description
6,359	Δ4 bp	5.3	101/777 nt[a]	*yaaA* ←	peroxide resistance protein, lowers intracellular iron
31,922	G→T	5.8	G369V (GGT→GTT)	*carB* →	carbamoyl phosphate synthase large subunit
76,053	T→A	5.8	E433D (GAA→GAT)	*leuC* ←	3-isopropylmalate dehydratase large subunit
85,212	T→C	5.1	L233P (CTG→CCG)	*cra* →	transcriptional repressor-activator for carbon metabolism
246,250	G→A	6.4	308/1713 nt	*lfhA* ←	pseudogene, flagellar system protein, promoterless fragment
381,062	insertion of IS5 into *yaiS*	44.3	insertion in the antisense direction at 16/558 of *yaiS*, with duplication of nts 13-16 of *yaiS* following the insertion	*yaiS* ←	putative deacetylase
728,279	G→A	5.4	A75T (GCC→ACC)	*astA* →	arginine succinyltransferase
850,006	C→T	13.3	R633H (CGC→CAC)	*rsxC* ←	SoxR iron-sulfur cluster reduction factor component

996,175	C→T	5.1	R144W (CGG→TGG)	*ddpX* →	D-Ala-D-Ala dipeptidase
1,178,452	C→T	5.7	A75T (GCG→ACG)	*ycjU* ←	β-phosphoglucomutase
1,195,051	Δ1 bp	5.3	1029/1488 nt	*puuC* ←	γ-glutamyl-γ-aminobutyr-aldehyde dehydrogenase; succinate semialdehyde dehydrogenase
1,406,900	G→A	6.9	G18G (GGC→GGT)	*fabG* ←	3-oxoacyl-[acyl-carrier-pr otein] reductase
1,430,870	C→T	6.4	V199V (GTG→GTA)	*yceH* ←	UPF0502 family protein
1,482,549	C→T	5.4	A94V (GCG→GTG)	*rutA* →	pyrimidine monooxygenase
1,484,978	C→T	5.2	A155V (GCG→GTG)	*rutD* →	putative aminoacrylate hydrolase
1,568,331	C→A	5.0	A346E (GCG→GAG)	*asnS* →	asparaginyl tRNA synthetase
1,582,599	G→A	5.5	L49L (CTG→TTG)	*smtA* ←	putative *S*-adenosyl-L-methionine-dependent methyltransferase
1,669,431	T→C	6.3	F164S (TTT→TCT)	*ybjJ* →	putative transporter
1,736,878	A→G	5.1	G36G (GGT→GGC)	*moaE*←	molybdopterin synthase, large subunit
1,811,088	C→A	5.1	G10C (GGC→TGC)	*ybgL* ←	UPF0271 family protein
1,933,140	G→A	5.1	L281L (CTG→TTG)	*entS* ←	enterobactin exporter, iron-regulated
2,386,910	C→T	10.0	S426S (TCG→TCA)	*nuoL* ←	NADH:ubiquinone oxidoreductase, membrane subunit L
2,499,192	C→T	5.0	P194P (CCG→CCA)	*ypdF* ←	aminopeptidase
2,534,893	G→T	9.1	A214E (GCG→GAG)	*cysU* ←	sulfate/thiosulfate ABC transporter inner membrane subunit
2,570,656	C→T	5.9	R248H (CGT→CAT)	*maeB* ←	NADP+-dependent malate dehydrogenase
2,632,084	G→A	7.7	A74V (GCG→GTG)	*yfgM* ←	putative anti-RcsB factor
2,638,219	C→T	6.1	A710T (GCG→ACG)	*pbpC* ←	peptidoglycan glycosyltransferase
2,824,879	T→A	5.4	E144D (GAA→GAT)	*norR* ←	DNA-binding dual transcriptional activator
3,079,435	Δ1 bp	6.0	+12/-292[b]	*yqgC* → / → *met K*	YqgC: uncharacterized protein; MetK: *S*-adenosylmethionine synthetase

3,321,484	insertion of IS*4* into *greA*	100.0	insertion in the antisense direction at 254/477 of *greA*, with duplication of nts 243-253 of *greA* following the insertion	*greA* ←	transcript cleavage factor
3,325,874	G→A	7.8	-118/+9	*yhbE* ← / ← *rpmA*	YhbE: putative transporter; RpmA: 50S ribosomal subunit protein L27
3,392,233	G→A	5.9	P256L (CCG→CTG)	*mreC* ←	cell shape determining protein
3,543,014	C→T	5.1	P26P (CCG→CCA)	*malQ* ←	4-α-glucanotransferase (amylomaltase)
3,688,150	C→G	6.7	G37R (GGC→CGC)	*bcsA* ←	cellulose synthase, catalytic subunit
3,808,881	Δ82 bp	100.0	deletion in *rph* increases expression of downstream gene *pyrE*	*rph* ←	truncated RNase PH
3,917,043	G→A	5.4	A538V (GCG→GTG)	*mnmG* ←	5-carboxymethyl-aminomethyl-uridine-tRNA synthase subunit
3,996,293	C→A	5.1	-78/+17	*yigG* ← / ← *rarD*	YigG: inner membrane protein; RarD: putative transporter
4,022,340	C→T	5.5	A552T (GCT→ACT)	*fadB* ←	multifunctional enoyl-CoA hydratase, 3-hydroxyacyl-CoA epimerase, Δ^3-*cis*- Δ^2-*trans*-enoyl-CoA isomerase, L-3-hydroxyacyl-CoA dehydrogenase
4,312,846	C→A	5.5	186/426 nt	*phnE* ←	defective phosphonate/phosphate ABC transporter subunit
4,543,660	C→T	5.8	A76T (GCT→ACT)	*yjiH* ←	gate family protein

Generation 1006

position	mutation	freq (%)	annotation	gene	description
28,061	G→T	7.9	E257 (GAA→TAA)	*rihC* →	ribonucleoside hydrolase 3
33,802	Δ132 bp	43.3	deletion in the regulatory domain of CarB	*carB* →	carbamoyl phosphate synthetase large subunit
214,624	C→T	5.6	L213L (CTG→CTA)	*proS* ←	prolyl-tRNA synthetase

381,062	insertion of IS5 into *yaiS*	89.4	insertion in the antisense direction at 16/558 of *yaiS*, with duplication of nts 13-16 of *yaiS* following the insertion	*yaiS* ←	putative deacetylase
412,382	G→A	8.3	G109S (GGC→AGC)	*alkA* →	3-methyl-adenine DNA glycosylase II
804,218	T→C	6.1	+328/-127	*fumD* → / →*ydhY*	YdhY: putative 4Fe-4S ferredoxin-type protein; FumD, fumarase D
908,012	G→A	5.1	733/765 nt	*ydfE* ←	Qin prophage, pseudogene,Phage or Prophage Related
937,098	C→A	5.5	T131T (ACG→ACT)	*ydeE* ←	putative transporter
1,482,124	C→T	5.2	-145/-86	*rutR* ← / → *rutA*	RutA: pyrimidine monooxygenase; RutR; DNA-binding transcriptional dual regulator
1,499,109	G→A	6.3	P304L (CCA→CTA)	*torT* ←	periplasmic trimethylamine-N-oxide binding protein associated with the TorTSR signal transduction system
1,646,149	G→A	5.6	A307T (GCA→ACA)	*poxB* →	pyruvate oxidase
1,741,717	T→C	5.6	Q346Q (CAA→CAG)	*uvrB* ←	excision nuclease subunit B
1,803,416	insertion of IS5 between *ybgD* and *gltA*	44.2	insertion in the antisense direction 66 nt upstream of *ybgD* and 321 nt downstream of *gltA* with duplication of 4 nts flanking the insertion	*ybgD* → / → *gltA*	YbgD: putative fimbrial protein; GltA: citrate synthase
2,007,659	A→T	6.1	F89I (TTC→ATC)	*ylbE* ←	NO-induced DUF1116 protein
2,0464,22	G→A	5.3	A508T (GCT→ACT)	*copA* →	copper transporter
2,298,989	Δ1 bp	5.1	906/1647 nt	*mqo* ←	malate:quinone oxidoreductase
2,368,331	G→A	5.5	T258M (ACG→ATG)	*menC* ←	*o*-succinylbenzoyl-CoA synthase
2,495,120	C→A	8.3	Q284K (CAG→AAG)	*ypdC* →	putative DNA-binding protein

2,840,954	C→T	7.0	V437I (GTC→ATC)	*hycC* ←	hydrogenase 3, membrane subunit
2,940,953	G→A	5.5	A360V (GCC→GTC)	*amiC* ←	*N*-acetylmuramoyl-L-alanine amidase C
2,955,700	G→A	5.2	A29V (GCG→GTG)	*ppdC* ←	prepilin-type N-terminal cleavage/methylation domain-containing protein PpdC
3,186,739	A→T	6.6	T285T (ACA→ACT)	*yqiK* →	flotillin family inner membrane protein
3,321,484	insertion of IS*4* into *greA*	93.2	insertion in the antisense direction at 254/477 of *greA*, with duplication of nts 243-253 of *greA* following the insertion	*greA* ←	transcription elongation factor
3,353,077	G→A	5.4	G287S (GGC→AGC)	*gltD* →	glutamate synthase subunit D
3,713,400	A→T	6.7	+117/+70	*cspA* → / ← *hokA*	CspA: cold-shock protein, RNA chaperone; HokA: small toxic polypeptide
3,808,881	Δ82 bp	100.0	deletion in *rph* increases expression of downstream gene *pyrE*	*rph* ←	truncated RNase PH
3,911,328	C→T	5.5	-41/+10	*atpG* ← / ← *atpA*	AtpG: ATP synthase F_1 complex γ subunit; AtpA: ATP synthase F_1 complex α subunit
4,117,616	C→G	5.7	-132/+52	*yiiX* ← / ← *metJ*	YiiX: putative lipid binding hydrolase; MetJ:DNA-binding transcriptional repressor
4,145,863	Δ51 bp	32.7	deletion upstream of *argB*	*argB* →	*N*-acetylglutamate kinase
4,308,103	G→A	6.3	Q155* (CAG→TAG)	*phnK* ←	carbon-phosphorus lyase complex subunit

[a] For mutations within genes, (x/y nt) indicates the position at which a mutation occurs (x) in a gene that is y nucleotides long.

[b]For mutations in intergenic regions, (x/y) indicates the distance to the genes upstream and downstream of the mutation.

Table S2. PCR primers used in this work.*

Name	sequence (5'⟶3')	use
1357	CGTGCAGGCGATTGATAA	forward primer for *proA* qPCR
1358	CTGTTCACGGCACAGTTT	reverse primer for *proA* qPCR
1359	CGTAGATCTGACGGTGAATTT	forward primer for *gyrB* qPCR
1360	CGTTGGTGTTTCGGTAGTA	reverse primer for *gyrB* qPCR
1361	CCCGTGGCTGAAAGTTAAA	forward primer for *icd* qPCR
1362	CAGGTTCATACAGGCGATAAC	reverse primer for *icd* qPCR
r02	TAGTCCGTCTGCATCGCATC	reverse primer for amplification of Clade 1 new junction between *frsA* and *mdtD*
r03	AGCAAGACAATGCAGGTGGC	forward primer for amplification of Clade 1 new junction between *frsA* and *mdtD*
r05	AAGGTCTTCCAGAGCGGTG	forward primer to detect new junction due to genomic rearrangement in parental strain AM187
r06	GCATCGAGCGCGTTTTATGG	reverse primer to detect new junction due to genomic rearrangement in parental strain AM187
r07	TTTTACCACCAGCGTCTGGC	reverse primer for amplification of Clade 2 new junction / identification of 110 kb deletion in Clade 1
r08	CTCATTCGCAAACGCAGTCG	forward primer for amplification of Clade 2 new junction / identification of 110 kb deletion in Clade 1
r09	ATTACCCAGTCGAACCCACG	reverse primer to detect insertion of IS*5* into *yaiS* / identification of 110 kb deletion in Clade 1
r10	AATGATGTCTTCCAGGGCGG	forward primer to detect insertion of IS*5* into *yaiS*
r12	CCGGCGTTTTGATGACCAC	forward primer to detect insertion of IS*4* into *greA*
r13	TGAGCATGGCGACCTGAAAG	reverse primer to detect insertion of IS*4* into *greA*
r14	AGCCGGTTCTATGATTGGCG	forward primer for amplification of Clade 3 new junction
r15	TCTGCTGTAAGTTGCAGGCG	reverse primer for amplification of Clade 3 new junction
r16	CCTGAGCAAAGACCCCAACG	forward primer for amplification of Clade 1b new 4.9 kb amplification junction (A4)
r17	CTTTGGTCTGCGTCCATCGC	reverse primer for amplification of Clade 1b new 4.9 kb amplification junction (A4)
r18	GTTCCGTTCGTCTCCAAAGC	forward primer to detect deletion of 132 bp in *carB*

r19	CACCGTCGATAAGTGTGAGC	reverse primer to detect deletion of 132 bp in *carB*
r20	GACATAGCGTTGGCTACCCG	forward primer to detect deletion of 51 bp upstream of *argB*
r21	CAGCCCTTTCATCAGCTCATCC	reverse primer to detect deletion of 51 bp upstream of *argB*
r22	GCCATTAGTGGGGCCTATAC	forward primer for amplification of Clade 5 new junction between *crl* and *yegI*
r23	GGTAAACGAGCGTACGGATG	reverse primer for amplification of Clade 5 new junction between *crl* and *yegI*
r24	gaacgccggaTGTAATAAAAGCGTAAACAACTGCCGCTAGGC	forward primer for amplification of a fragment from 150 bp upstream of the *proBA** operon through the first 171 bp of *proA** for construction of the *proBA*-gfp* fusion reporter in pACYC177 by Gibson assembly; this fragment contains the M2 promoter mutation
r25	ctttactcatGCCATTGGCTCGCGCGTCAG	reverse primer for amplification of a fragment from 150 bp upstream of the *proBA** operon through the first 171 bp of *proA** for construction of the *proBA*-gfp* fusion reporter in pACYC177 by Gibson assembly; this fragment contains the M2 promoter mutation
r26	agccaatggcATGAGTAAAGGAGAAGAACTTTTC	forward primer for amplification of *gfp* from pGRG36-Kn-PA1-GFP, including a T7 terminator sequence after the stop codon, for construction of the *proBA*-gfp* fusion reporter in pACYC177 by Gibson assembly
r27	tctaggatccCAAAAAACCCCTCAAGACCC	reverse primer for amplification of *gfp* from pGRG36-Kn-PA1-GFP, including a T7 terminator sequence after the stop codon, for construction of the *proBA*-gfp* fusion reporter in pACYC177 by Gibson assembly
r28	gggttttttgGGATCCTAGAGCGCACGAATGAGG	forward primer for amplification of the pACYC vector backbone for construction of the *proBA*-gfp* fusion reporter in pACYC177 by Gibson assembly
r29	ttttattacaTCCGGCGTTCAGCCTGTGCC	reverse primer for amplification of the pACYC vector backbone for construction of the *proBA*-gfp* fusion reporter in pACYC177 by Gibson assembly
r30	GCCACTAAGTCACCCTTAAGTAC	forward primer for amplifying *greA* upstream homology region for

		Δ*greA*::*tetR*-*ccdB*-*cat* construct and amplifying *greA*::IS4 with flanking homology regions
r31	cggccagtgaatccgtaatgCCTCTTGAATATTCCTGATAGGGC	reverse primer for amplifying *greA* upstream homology region for Δ*greA*::*tetR*-*ccdB*-*cat* construct
r32	CATTACGGATTCACTGGCCG	forward primer for amplifying *tetR*-*ccdB*-*cat* from pDLM3
r33	CGGTAAATAGCTTGCCTGCTC	reverse primer for amplifying *tetR*-*ccdB*-*cat* from pDLM3
r34	gagcaggcaagctatttaccgACCCAATACTCAAGATGTTGATG	forward primer for amplifying *greA* downstream homology region for Δ*greA*::*tetR*-*ccdB*-*cat* construct
r35	CATGGGGATAACTCATCGTAAC	reverse primer for amplifying *greA* downstream homology region for Δ*greA*::*tetR*-*ccdB*-*cat* construct and amplifying *greA*::IS4 with flanking homology regions

*Lowercase letters indicate overlap regions for Gibson assembly.

Table S3. Genotypes of 40 colonies isolated from population 2 at generation 227. PCR data supporting the table entries are shown in Supplementary Figs. S3 and S4.

clone #	glycerol stock	Clade 1 amplification (indicates A1, A2 and/or A3)	Insertion of IS*5* into *yaiS* (indicates A2)	No insertion of IS*5* into *yaiS* (indicates A1)	110 kb deletion in A2 (indicates A3)	Clade 2 amplification	Clade 3 amplification	Mutation upstream of *proA**	Clade 5 amplification
C1	AM500	x	x	x	x				
C2	AM501	x	x	x	x				
C3	AM502	x	x	x	x				
C4	AM503						x		
C5	AM504	x	x	x	x				
C6	AM505	x	x	x	x				
C7	AM506						x		
C8	AM507	x	x	x	x				
C9	AM508	x	x	x	x				
C10	AM509							x	
C11	AM510	x	x	x	x				
C12	AM511						x		
C13	AM512	x	x	x	x				
C14	AM513							x	
C15	AM514	x	x	x	x				
C16	AM515					x			
C17	AM516	x	x	x	x				
C18	AM517					x			
C19	AM518	x	x	x	x				
C20	AM519	x	x	x	x				
C21	AM520	x	x	x	x				
C22	AM521	x	x	x	x				
C23	AM522	x	x	x	x				
C24	AM523								x
C25	AM524	x	x	x	x				
C26	AM525							x	
C27	AM526	x	x	x	x				
C28	AM527	x	x	x	x				
C29	AM528	x	x	x	x				
C30	AM529					x			
C31	AM530	x	x	x	x				
C32	AM531					x			
C33	AM532	x	x	x	x				
C34	AM533					x			
C35	AM534	x	x	x	x				
C36	AM535	x	x	x	x				
C37	AM536	x	x	x	x				
C38	AM537						x		
C39	AM538	x	x	x	x				
C40	AM539	x	x	x	x				
freq:		67.5%	67.5%	67.5%	67.5%	12.5%	10%	7.5%	2.5%

Clade 1
Clade 2
Clade 3
Clade 4
Clade 5

Table S4. Genotypes of 37 colonies isolated from population 2 at generation 669. PCR data supporting the table entries are shown in Supplementary Fig. S5.

clone #	glycerol stock	Clade 1 amplification (indicates A1, A2 and/or A3)	insertion of IS*5* into *yaiS* (indicates A2)	no insertion of IS*5* into *yaiS* (indicates A1)	110 kb deletion in A2 (indicates A3)	132 bp deletion in *carB*	Clade 1 amplification A4	insertion of IS*4* into *greA*
C1	AM540						x	x
C2	AM541	x	x	x	x	x		
C3	AM542						x	x
C4	AM543						x	x
C5	AM544						x	x
C6	AM545						x	x
C7	AM546	x	x	x	x	x		
C8	AM547						x	x
C9	AM548						x	x
C10	AM549	x	x		x	x		
C11	AM550						x	x
C12	AM551						x	x
C13	AM552						x	x
C14	AM553						x	x
C15	AM554						x	x
C16	AM555						x	x
C17	AM556						x	x
C18	AM557						x	x
C21	AM560	x	x	x	x	x		
C22	AM561						x	x
C23	AM562						x	x
C24	AM563						x	x
C25	AM564						x	x
C26	AM565						x	x
C28	AM567						x	x
C29	AM568						x	x
C30	AM569						x	x
C31	AM570						x	x
C32	AM571						x	x
C33	AM572						x	x
C34	AM573						x	x
C35	AM574	x		x	x		x	x
C36	AM575	x		x			x	x
C37	AM576						x	x
C38	AM577	x	x	x	x	x		
C39	AM578						x	x
C40	AM579						x	x
	freq:	18.9%	13.5%	16.2%	16.2%	13.5%	86.5%	86.5%

lineage 1a.1

lineage 1b.1

lineage 1b.2

Table S5. Genotypes of 40 colonies isolated from population 2 at generation 1006. PCR data supporting the table entries are shown in Supplementary Fig. S6.

clone #	glycerol stock	Clade 1 amplification (indicates A1, A2 and/or A3)	insertion of IS*5* into *yaiS* (indicates A2)	no insertion in *yaiS* (indicates wt sequence or A1)	110 kb deletion in A2 (indicates A3)	loss of full *yaiS* sequence (indicates only A3 present)	132 bp deletion in *carB*	A4	insertion of IS*4* into *greA*	deletion of 51 bp upstream of *argB*
C1	AM580			x				x	x	x
C2	AM581			x				x	x	x
C3	AM582			x				x	x	x
C4	AM583			x				x	x	x
C5	AM584	x			x		x		x	
C6	AM585			x				x	x	x
C7	AM586			x				x	x	x
C8	AM587	x			x		x		x	
C9	AM588	x	x	x	x		x			
C10	AM589	x	x	x	x		x			
C11	AM590			x				x	x	
C12	AM591	x			x	x	x		x	
C13	AM592	x			x		x		x	
C14	AM593	x			x		x		x	
C15	AM594			x				x	x	x
C16	AM595			x				x	x	x
C17	AM596	x	x	x	x		x			
C18	AM597	x			x	x	x		x	
C19	AM598			x				x	x	x
C20	AM599			x				x	x	x
C21	AM600			x				x	x	x
C22	AM601			x				x	x	x
C23	AM602	x			x		x		x	
C24	AM603	x			x		x		x	
C25	AM604			x				x	x	x
C26	AM605			x				x	x	
C27	AM606	x			x	x	x		x	
C28	AM607			x				x	x	x
C29	AM608			x				x	x	
C30	AM609	x			x	x	x		x	
C31	AM610	x	x	x	x		x			
C32	AM611	x	x	x	x		x			
C33	AM612	x			x		x		x	
C34	AM613	x			x		x		x	
C35	AM614			x				x	x	x
C36	AM615	x	x	x	x		x			
C37	AM616	x			x		x		x	
C38	AM617	x			x		x		x	
C39	AM618			x				x	x	x
C40	AM619			x				x	x	
	freq:	50%	15%	65%	50%	10%	50%	50%	85%	40%

lineage 1a.1

lineage 1a.2

lineage 1b.2

lineage 1b.3

Supplementary Table S6. Plasmids used in this work.

**Name**	**description**	**source**
pACYC117	low-copy number *E. coli* expression vector; (amp^R)	ATCC
pDLM3	vector encoding *tetR*-*ccdB*-*cat* selection-counterselection markers module (chl^R)	PMID: 31589608 (Bossi, et al. 2019)
pGRG36-Kn-PA1-GFP	low-copy number vector encoding mini-Tn7 transposase system for *gfp* integration at the Tn7 *att* site and pSC101 origin with temperature-sensitive Rep101 (kan^R)	PMID: 32060029 (Yang, et al. 2020)
pGRG36-Amp-GFP	low-copy number vector encoding mini-Tn7 transposase system for GFP integration at Tn7 *att* site and pSC101 origin with temperature-sensitive Rep101 (amp^R)	this study
pWTproA57-gfp	translational fusion reporter plasmid encoding the *proBA** operon with the M2 promoter mutation through the first 57 codons of *proA** fused to *gfp* in pACYC177	this study; the M2 promoter mutation is described in (Kershner, et al. 2016)
pClade4mutproA57-gfp	translational fusion reporter plasmid encoding the *proBA** operon with the M2 promoter mutation and the G to A mutation at -4 relative to the start codon of *proA** found in Clade 4 through the first 57 codons of *proA** fused to *gfp* in pACYC177	this study; the M2 promoter mutation is described in (Kershner, et al. 2016)

Table S7. Amplification junctions in Clades 1, 2, 3 and 5.

amplicon	gene	sequence (5'→3')
Clade 1 A1, A2 and A3	*frsA*	GCGGCTACGTTGTACAACAT TGCCGCCTATCCTCATCTGA
	new junction	CAGCAACAGCCCGGCGATAG TGCCGCCTATCCTCATCTGA
	mdtD	CAGCAACAGCCCGGCGATAG TGACGCCGATACTCATCGAC
Clade 1 A4	*phoE*	GATACGTCATGCCAACCGCG ACAATATCATCATTATTAAT
	new junction	TTATCATTCTCCCACCAAAA ACAATATCATCATTATTAAT
	thrW/ykfN	TTATCATTCTCCCACCAAAA AATTATCTTAATGTAACAGC
Clade 2	*phoE/proB*	TCTTAAAATGTGGTAATTTA TTAAATCTGTAATAAAAGCG
	new junction	GGGACTTGTTCGCACCTTCC TTAAATCTGTAATAAAAGCG
	IS*5*	GGGACTTGTTCGCACCTTCC GGAGGCGTTATGAGCTGGCG
Clade 3	IS1	TTCATCGCATGGACAATACG GGTGATGCTGCCAACTTACT
	new junction	ATGTGAAATATCGTTTTGTT GGTGATGCTGCCAACTTACT
	yahK	ATGTGAAATATCGTTTTGTT ATCGATAATCGCACACTAAC
Clade 5	IS1	TCTCAAGCGTACGTATTGTC GGTAATGACTCCAACTTATT
	new junction	AGTATTTACATCGACAGGTG GGTAATGACTCCAACTTATT
	yegI	AGTATTTACATCGACAGGTG AATTGACCACTCTTGGCCGT

Table S8. Genes that were lost in lineage 1a.2 due to the 110 kb deletion between two IS*3* elements.

Gene	Annotation
mmuP	*S*-methyl-L-methionine transporter
mmuM	homocysteine *S*-methyltransferase
afuC	CP4-6 prophage; ABC transporter ATP-binding protein AfuC
afuB	CP4-6 prophage; ABC transporter membrane protein AfuB
insB2	IS1 protein InsB
InsA2	IS1 protein InsA
insI2	pseudogene
insX	pseudogene
yagB	orphan antitoxin YagB
yagA	CP4-6 prophage; integrase core domain-containing protein YagA
yagE	CP4-6 prophage; putative 2-keto-3-deoxygluconate aldolase
yagF	CP4-6 prophage; D-xylonate dehydratase
yagG	putative D-xylonate transporter YagG
yagH	CP4-6 prophage; putative xylosidase/arabinosidase
yagI	CP4-6 prophage; DNA-binding transcriptional repressor XynR
argF	CP4-6 prophage; ornithine carbamoyltransferase ArgF
ykgS	CP4-6 prophage; protein YkgS
insB3	IS1 protein InsB
insA3	IS1 protein InsA
yagJ	CP4-6 prophage; protein YagJ
yagK	CP4-6 prophage; uncharacterized protein YagK
yagL	CP4-6 prophage; resolvase-like catalytic domain-containing protein YagL
yagM	CP4-6 prophage; protein YagM
yagN	CP4-6 prophage; protein YagN
intF	CP4-6 prophage; putative phage integrase
nc1	small noncoding RNA involved in nucleoid organization
yagP	putative LysR family substrate binding domain-containing protein YagP
paoD	molybdenum cofactor insertion chaperone for PaoABC
paoC	aldehyde dehydrogenase: molybdenum cofactor-binding subunit
paoB	aldehyde dehydrogenase, FAD-binding subunit
paoA	aldehyde dehydrogenase, Fe-S subunit
yagU	inner membrane protein that contributes to acid resistance
ykgJ	putative zinc- or iron-chelating domain-containing protein YkgJ
ecpE	putative fimbrial chaperone EcpE
ecpD	fimbrial adhesin EcpD
ecpC	putative fimbrial usher protein EcpC
ecpB	putative fimbrial chaperone EcpB
ecpA	common pilus major subunit

ecpR	DNA-binding transcriptional dual regulator MatA
ykgL	uncharacterized protein YkgL
ykgO	putative ribosomal protein
ykgM	putative ribosomal protein
ykgR	putative membrane protein YkgR
ykgP	putative oxidoreductase
eaeH	putative porin domain-containing protein EaeH
insE1	IS3 element protein InsE
insF1	IS3 element protein InsF
ykgA	putative DNA-binding transcriptional regulator YkgA
ykgQ	pseudogene
rclC	reactive chlorine species resistance protein C
rclB	DUF1471 domain-containing protein RclB
rclA	putative pyridine nucleotide-disulfide oxidoreductase RclA
rclR	DNA-binding transcriptional activator RclR
ykgE	putative lactate utilization oxidoreductase YkgE
ykgF	putative amino acid dehydrogenase with NAD(P)-binding domain and ferridoxin-like domain
ykgG	DUF162 domain-containing lactate utilization protein YkgG
ykgH	uncharacterized protein YkgH
betA	choline dehydrogenase
betB	betaine aldehyde dehydrogenase
betI	DNA-binding transcriptional repressor BetI
betT	choline:H(+) symporter
pdeL	DNA-binding transcriptional activator/c-di-GMP phosphodiesterase PdeL
yahB	putative LysR-type DNA-binding transcriptional regulator YahB
yahC	uncharacterized protein YahC
yahD	ankyrin repeat-containing protein YahD
yahE	DUF2877 domain-containing protein YahE
yahF	putative acyl-CoA synthetase YahF
yahG	DUF1116 domain-containing protein YahG
yahI	carbamate kinase-like protein YahI
yahJ	putative deaminase with metallo-dependent hydrolase domain
yahK	aldehyde reductase, NADPH-dependent
yahL	uncharacterized protein YahL
yahM	uncharacterized protein YahM
yahN	putative amino acid exporter
yahO	DUF1471 domain-containing protein YahO
prpR	DNA-binding transcriptional dual regulator PrpR
prpB	2-methylisocitrate lyase
prpC	2-methylcitrate synthase
prpD	2-methylcitrate dehydratase

prpE	propionyl-CoA synthetase
codB	cytosine transporter
codA	cytosine/isoguanine deaminase
cynR	DNA-binding transcriptional dual regulator CynR
cynT	carbonic anhydrase 1
cynS	cyanase
cynX	cyanate transporter
lacA	galactoside O-acetyltransferase
lacY	lactose permease
lacZ	beta-galactosidase
lacI	DNA-binding transcriptional repressor LacI
mhpR	DNA-binding transcriptional activator MhpR
mhpA	putative 3-(3-hydroxyphenyl)propanoate/3-hydroxycinnamate hydroxylase
mhpB	3-carboxyethylcatechol 2,3-dioxygenase
mhpC	2-hydroxy-6-ketonona-2,4-dienedioate hydrolase
mhpC	2-hydroxy-6-ketonona-2,4-dienedioate hydrolase
mhpD	2-hydroxypentadienoate hydratase
mhpF	acetaldehyde dehydrogenase (acylating)
mhpE	4-hydroxy-2-oxovalerate aldolase
mhpT	3-hydroxyphenylpropionate/3- hydroxycinnamate:H(+) symporter
yaiL	DUF2058 domain-containing protein YaiL
frmB	S-formylglutathione hydrolase FrmB
frmA	S-(hydroxymethyl)glutathione dehydrogenase
frmR	DNA-binding transcriptional repressor FrmR
yaiO	outer membrane protein YaiO
yaiX_2	putative acyltransferase, N-terminal fragment
insC1	IS2 insertion element repressor InsA
insD1	IS2 element protein
yaiX_1	putative acyltransferase, N-terminal fragment
yaiP	putative glucosyltransferase
yaiS	putative deacetylase YaiS

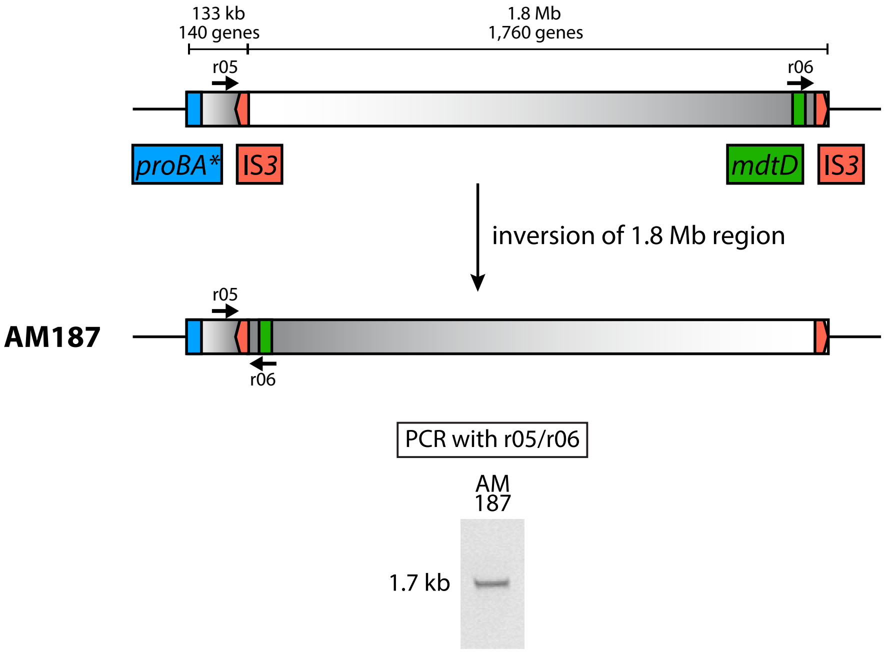

Fig. S1. A genomic rearrangement in the parental strain AM187 caused by inversion of a 1.8 Mb region flanked by IS*3* elements. The rearrangement was confirmed by long-read sequencing and by PCR with primers r05 and r06. Diagram is not to scale.

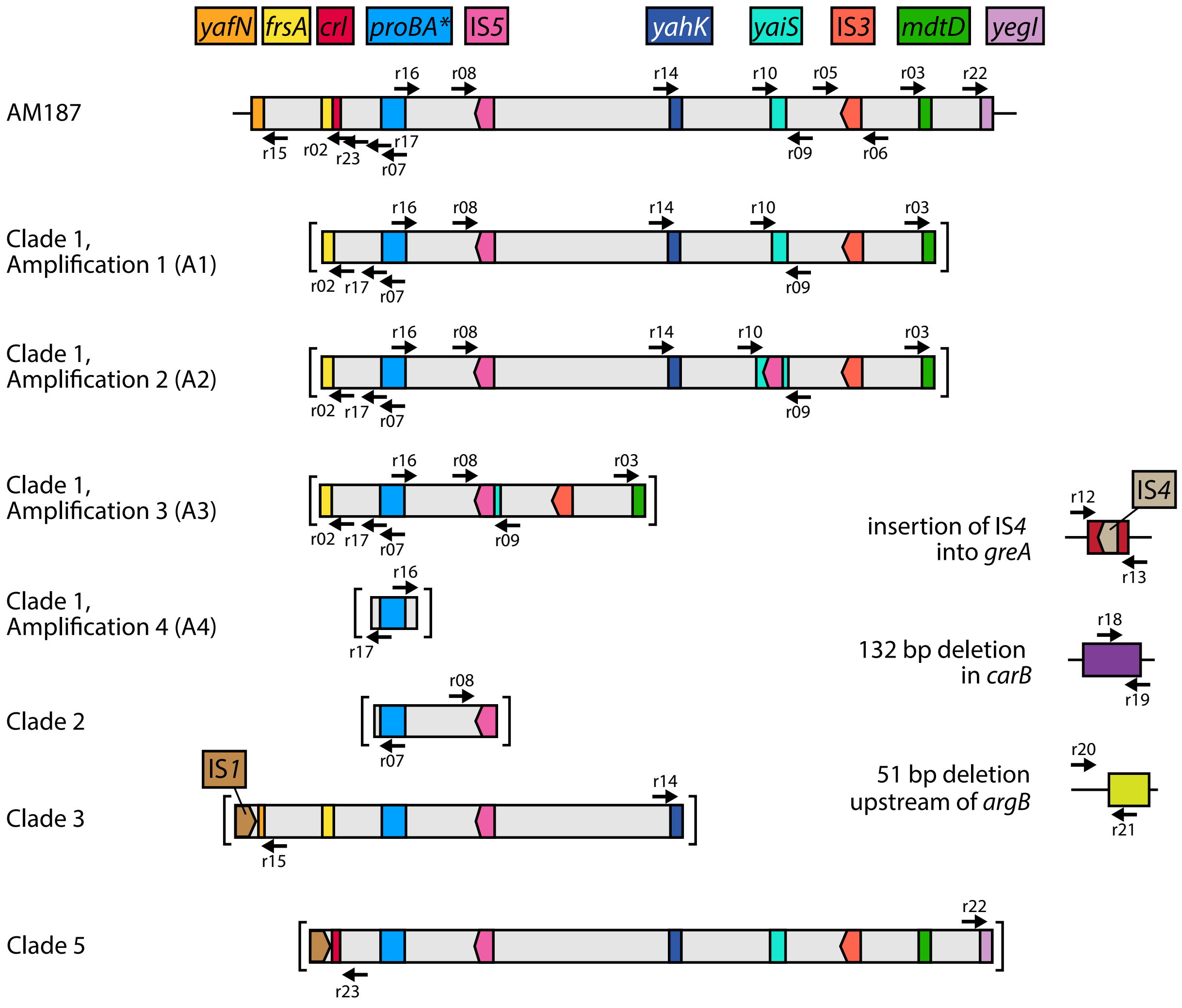

Fig. S2. Primers used to identify the genotypes of 40 colonies from population 2 at generations 227, 669 and 1006. Diagram is not to scale.

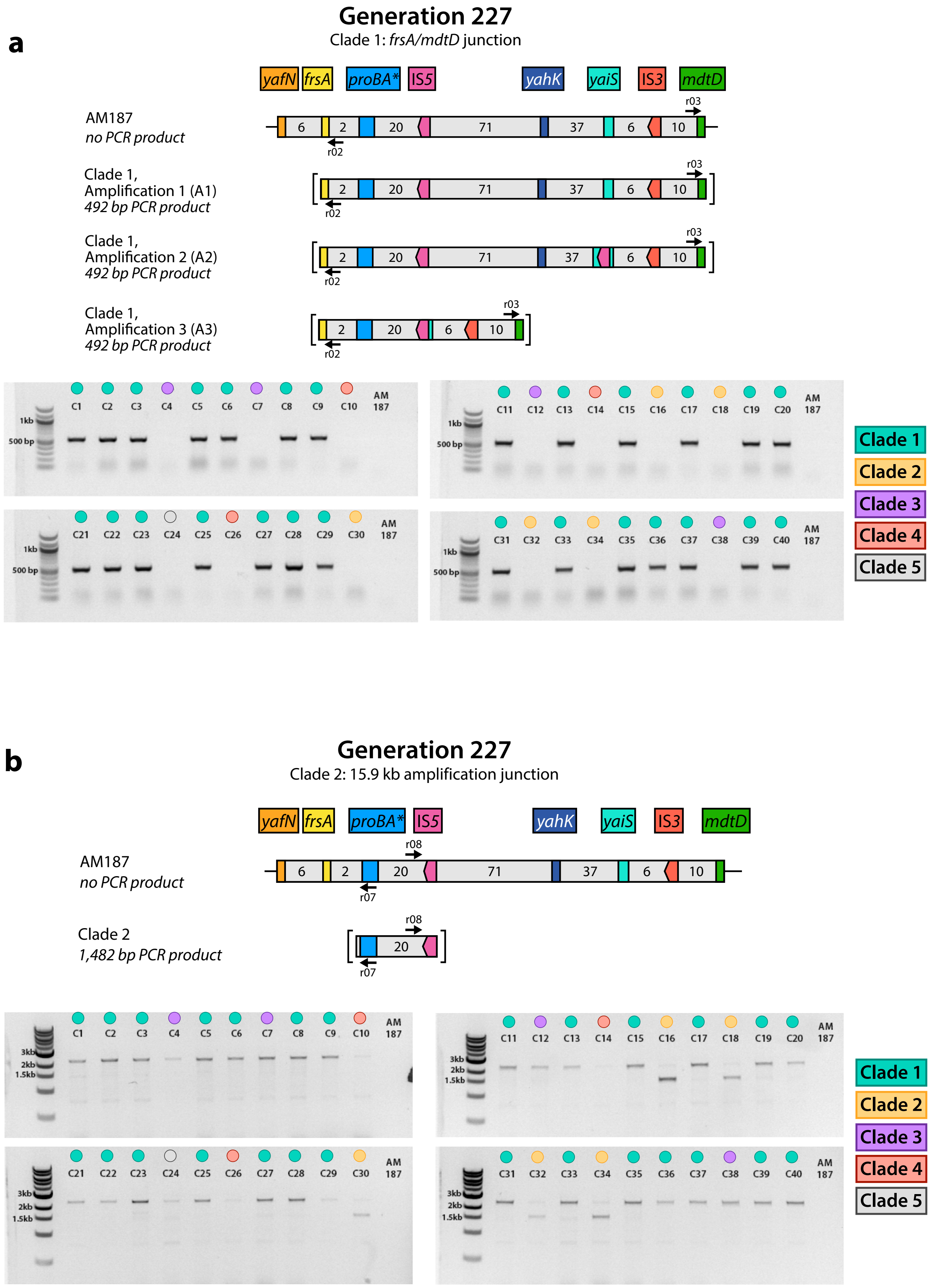
a
Generation 227
Clade 1: frsA/mdtD junction
yafN
frsA
proBA*
IS5
yahK
yaiS
IS3
mdtD
AM187
no PCR product
Clade 1,
Amplification 1 (A1)
492 bp PCR product
Clade 1,
Amplification 2 (A2)
492 bp PCR product
Clade 1,
Amplification 3 (A3)
492 bp PCR product
r02
r03
1kb
500 bp
AM 187
Clade 1
Clade 2
Clade 3
Clade 4
Clade 5
b
Generation 227
Clade 2: 15.9 kb amplification junction
AM187
no PCR product
Clade 2
1,482 bp PCR product
r07
r08
3kb
2kb
1.5kb

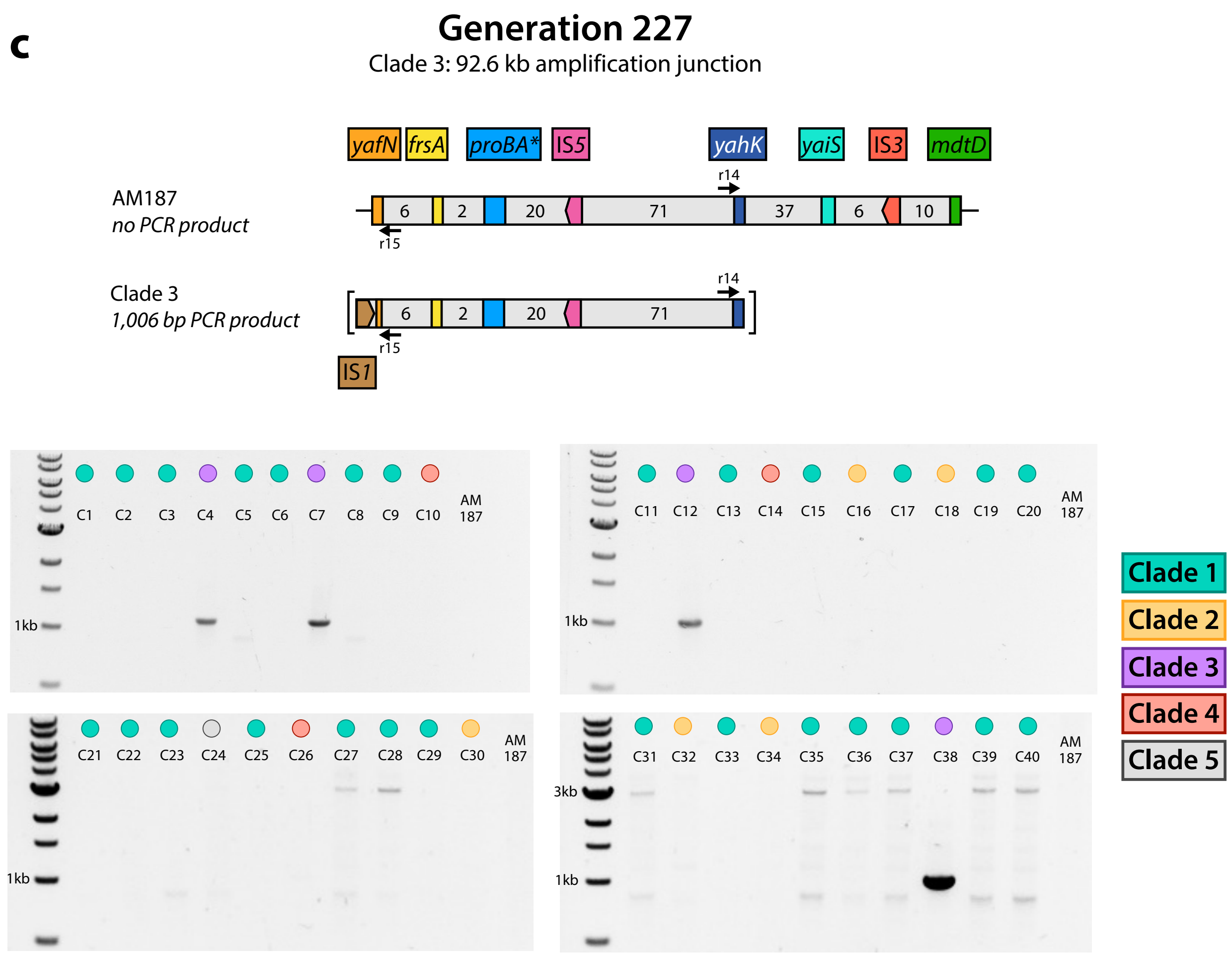

Fig. S3. PCR analysis of the amplified regions in 40 colonies isolated from population 2 at generation 227 with primers as indicated. Clades were identified based upon patterns of PCR products and/or whole-genome sequencing. **a**, Primers r02 and r03 amplify a 492 bp fragment in Clade 1 due to a new junction between *frsA* and *mdtD*. **b**, Primers r07 and r08 amplify a 1,482 bp fragment due to a new junction between a region upstream of the *proBA** operon and an IS*5* element. **c**, Primers r14 and r15 amplify a 1,006 bp fragment due to a new junction between an IS*1* element and *yahK*. The three Clade 4 colonies (C10, C14 and C25) did not have an amplified region. The 154 kb amplification in the Clade 5 colony (C23) was identified by whole-genome sequencing.

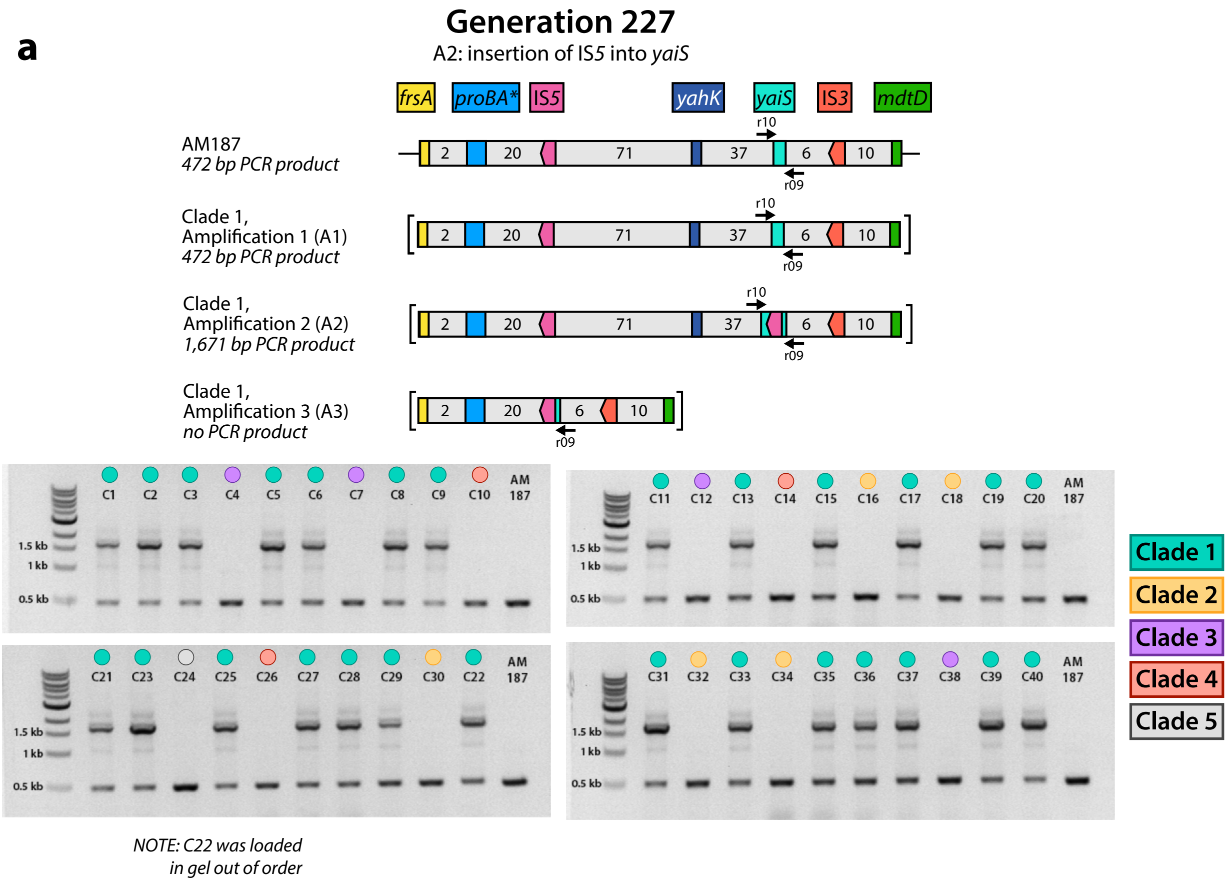
a
Generation 227
A2: insertion of IS5 into yaiS
frsA
proBA*
IS5
yahK
yaiS
IS3
mdtD
AM187
472 bp PCR product
Clade 1,
Amplification 1 (A1)
472 bp PCR product
Clade 1,
Amplification 2 (A2)
1,671 bp PCR product
Clade 1,
Amplification 3 (A3)
no PCR product
r10
r09
1.5 kb
1 kb
0.5 kb
AM 187
Clade 1
Clade 2
Clade 3
Clade 4
Clade 5
NOTE: C22 was loaded in gel out of order

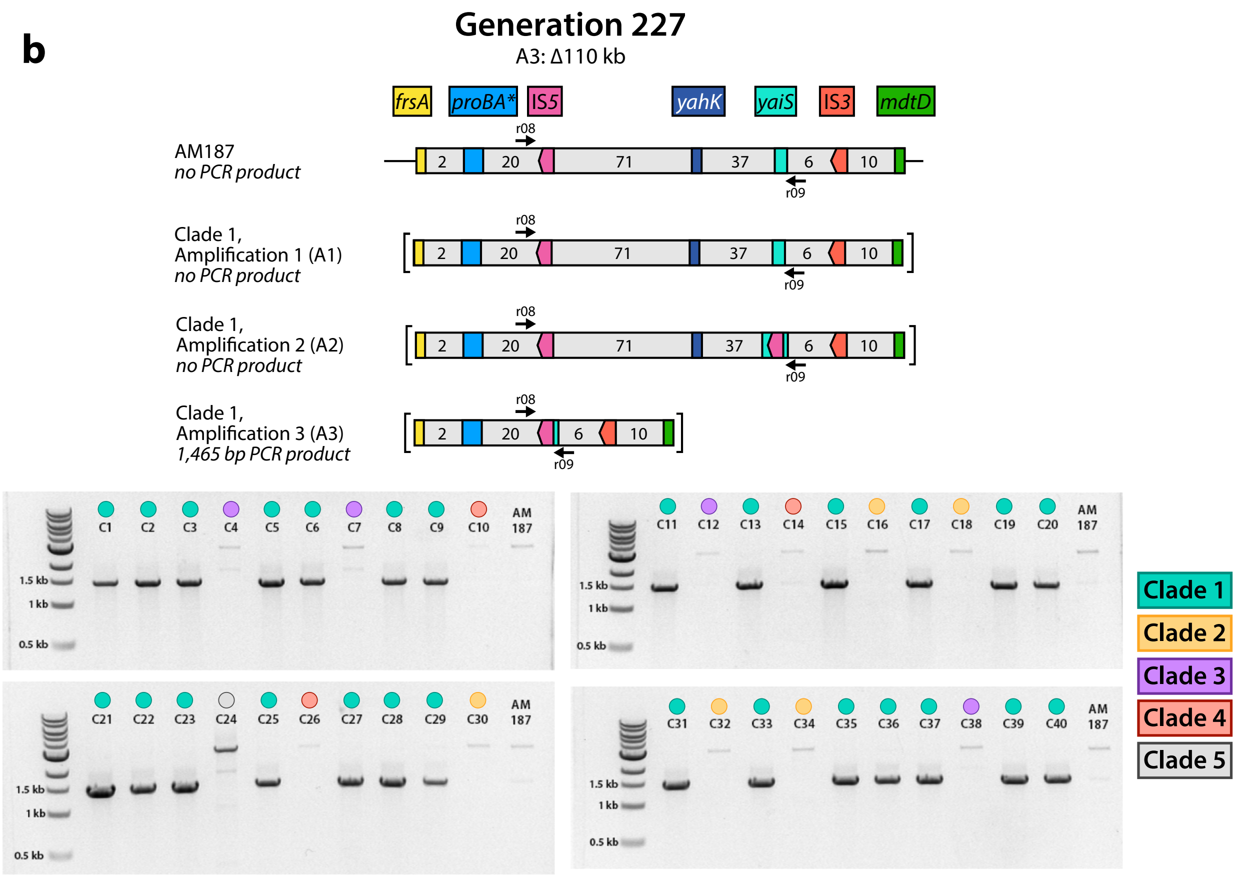

Fig. S4. PCR evidence for the simultaneous presence of amplicons A1, A2 and A3 in Clade 1 at generation 227. **a**, All Clade 1 colonies show bands consistent with insertion of IS5 into a copy of the initial amplicon A1, resulting in the presence of both A1 (no insertion, 472 bp product) and A2 (insertion, 1671 bp product). **b**, All Clade 1 colonies also have amplicon A3, which resulted from a 110 kb deletion between the two IS*5* elements (magenta) in amplicon A2. The 110 kb deletion includes 109 kb of genomic DNA and 1 kb of IS*5*.

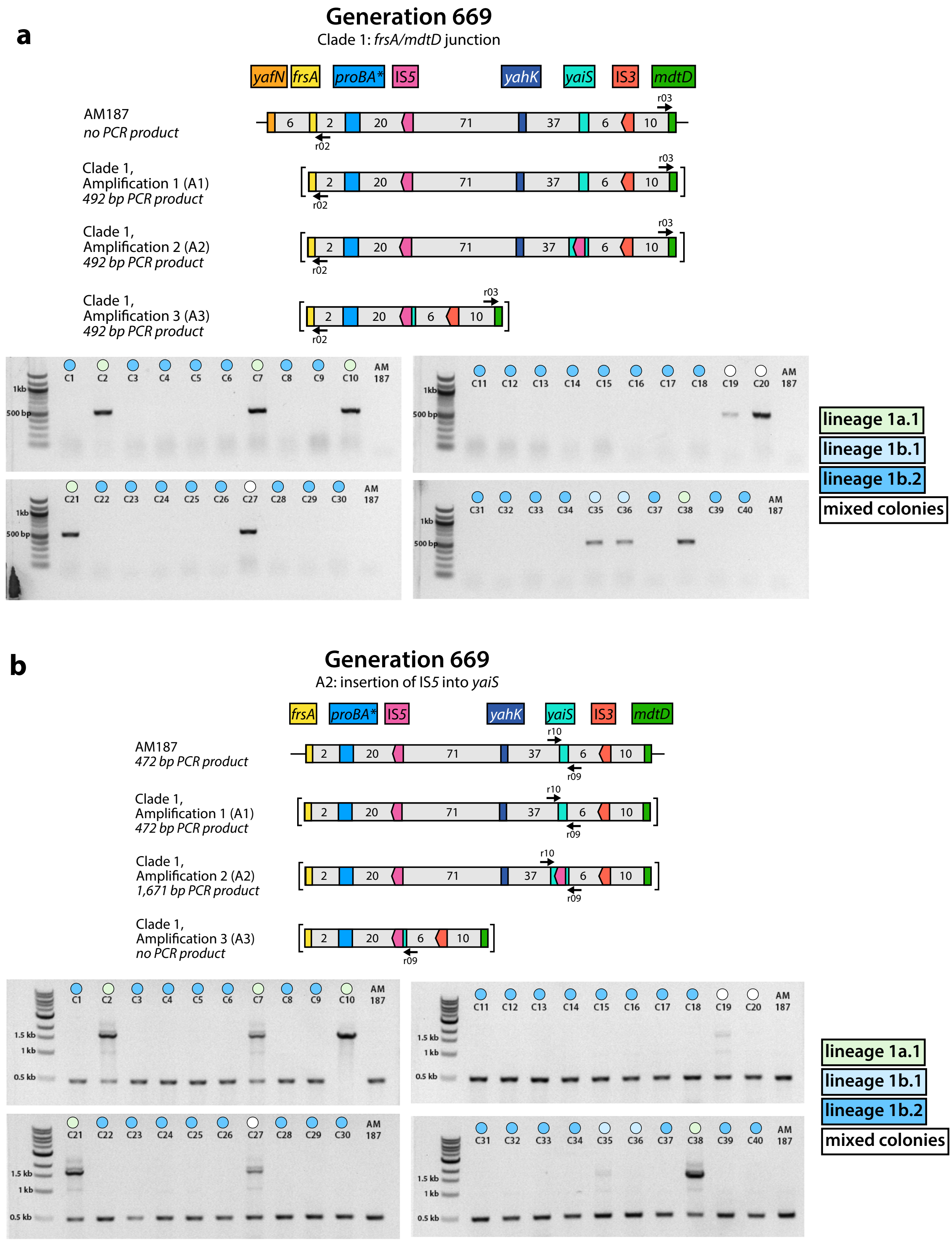
a
Generation 669
Clade 1: frsA/mdtD junction
yafN frsA proBA* IS5 yahK yaiS IS3 mdtD
AM187
no PCR product
Clade 1,
Amplification 1 (A1)
492 bp PCR product
Clade 1,
Amplification 2 (A2)
492 bp PCR product
Clade 1,
Amplification 3 (A3)
492 bp PCR product
r02
r03
1kb
500 bp
C1 C2 C3 C4 C5 C6 C7 C8 C9 C10 AM 187
C11 C12 C13 C14 C15 C16 C17 C18 C19 C20 AM 187
C21 C22 C23 C24 C25 C26 C27 C28 C29 C30 AM 187
C31 C32 C33 C34 C35 C36 C37 C38 C39 C40 AM 187
lineage 1a.1
lineage 1b.1
lineage 1b.2
mixed colonies
b
Generation 669
A2: insertion of IS5 into yaiS
frsA proBA* IS5 yahK yaiS IS3 mdtD
AM187
472 bp PCR product
Clade 1,
Amplification 1 (A1)
472 bp PCR product
Clade 1,
Amplification 2 (A2)
1,671 bp PCR product
Clade 1,
Amplification 3 (A3)
no PCR product
r09
r10
1.5 kb
1 kb
0.5 kb
C1 C2 C3 C4 C5 C6 C7 C8 C9 C10 AM 187
C11 C12 C13 C14 C15 C16 C17 C18 C19 C20 AM 187
C21 C22 C23 C24 C25 C26 C27 C28 C29 C30 AM 187
C31 C32 C33 C34 C35 C36 C37 C38 C39 C40 AM 187
lineage 1a.1
lineage 1b.1
lineage 1b.2
mixed colonies

c

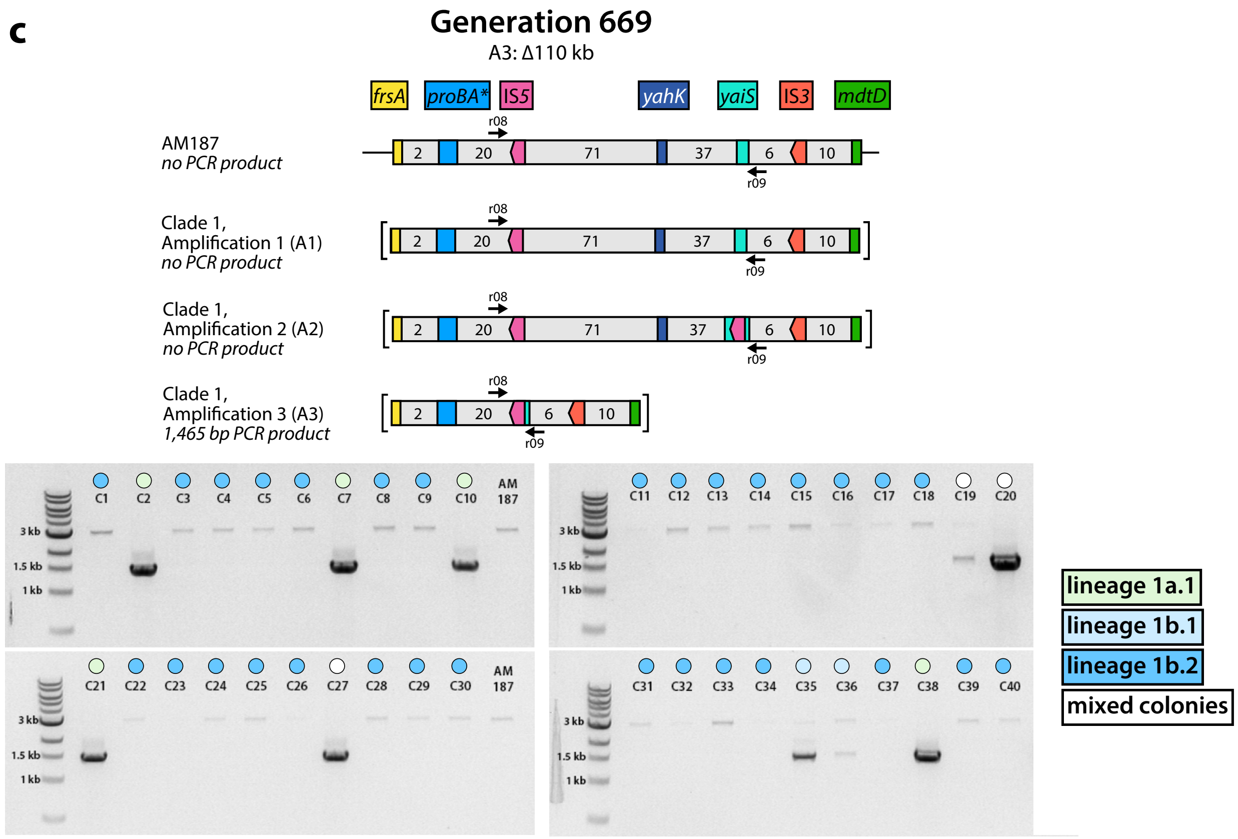

Generation 669
A3: Δ110 kb
frsA
proBA*
IS5
yahK
yaiS
IS3
mdtD
AM187
no PCR product
Clade 1,
Amplification 1 (A1)
no PCR product
Clade 1,
Amplification 2 (A2)
no PCR product
Clade 1,
Amplification 3 (A3)
1,465 bp PCR product
r08
r09
2
20
71
37
6
10
3 kb
1.5 kb
1 kb
C1 C2 C3 C4 C5 C6 C7 C8 C9 C10 AM 187
C11 C12 C13 C14 C15 C16 C17 C18 C19 C20
C21 C22 C23 C24 C25 C26 C27 C28 C29 C30 AM 187
C31 C32 C33 C34 C35 C36 C37 C38 C39 C40
lineage 1a.1
lineage 1b.1
lineage 1b.2
mixed colonies

d

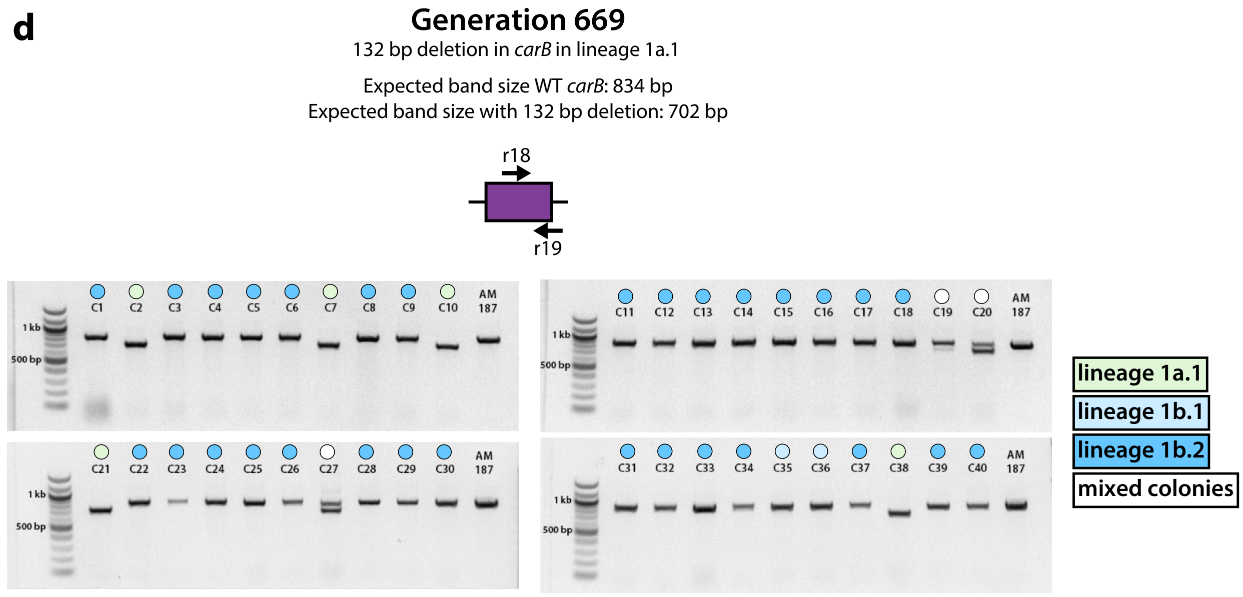

Generation 669
132 bp deletion in carB in lineage 1a.1
Expected band size WT carB: 834 bp
Expected band size with 132 bp deletion: 702 bp
r18
r19
1 kb
500 bp
C1 C2 C3 C4 C5 C6 C7 C8 C9 C10 AM 187
C11 C12 C13 C14 C15 C16 C17 C18 C19 C20 AM 187
C21 C22 C23 C24 C25 C26 C27 C28 C29 C30 AM 187
C31 C32 C33 C34 C35 C36 C37 C38 C39 C40 AM 187
lineage 1a.1
lineage 1b.1
lineage 1b.2
mixed colonies

e

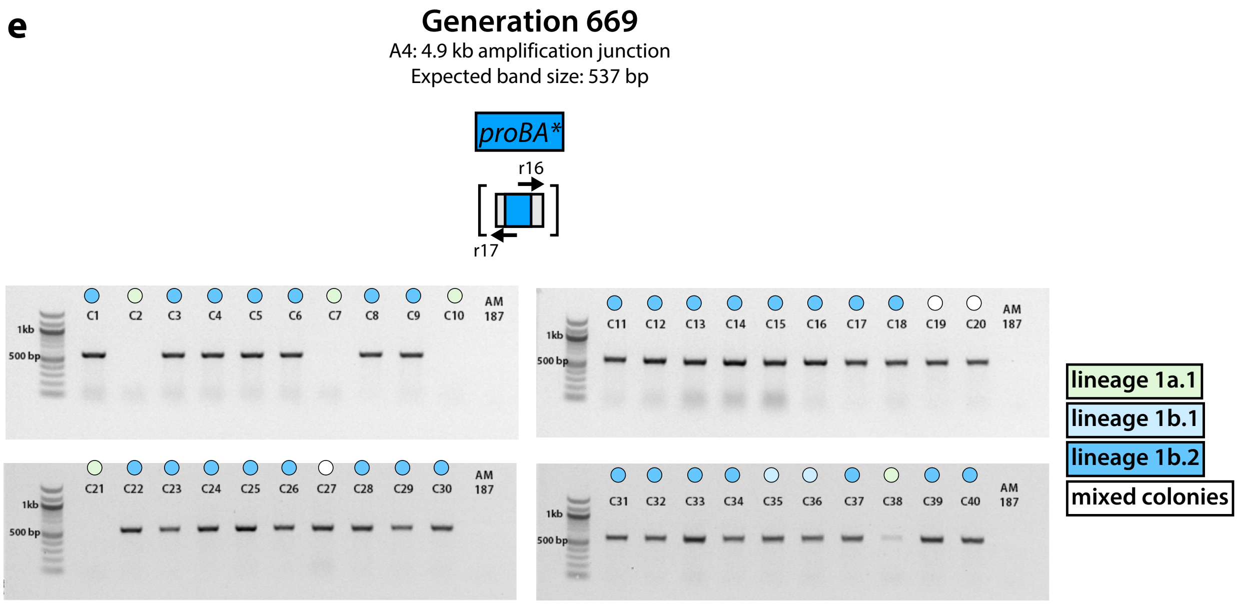

f

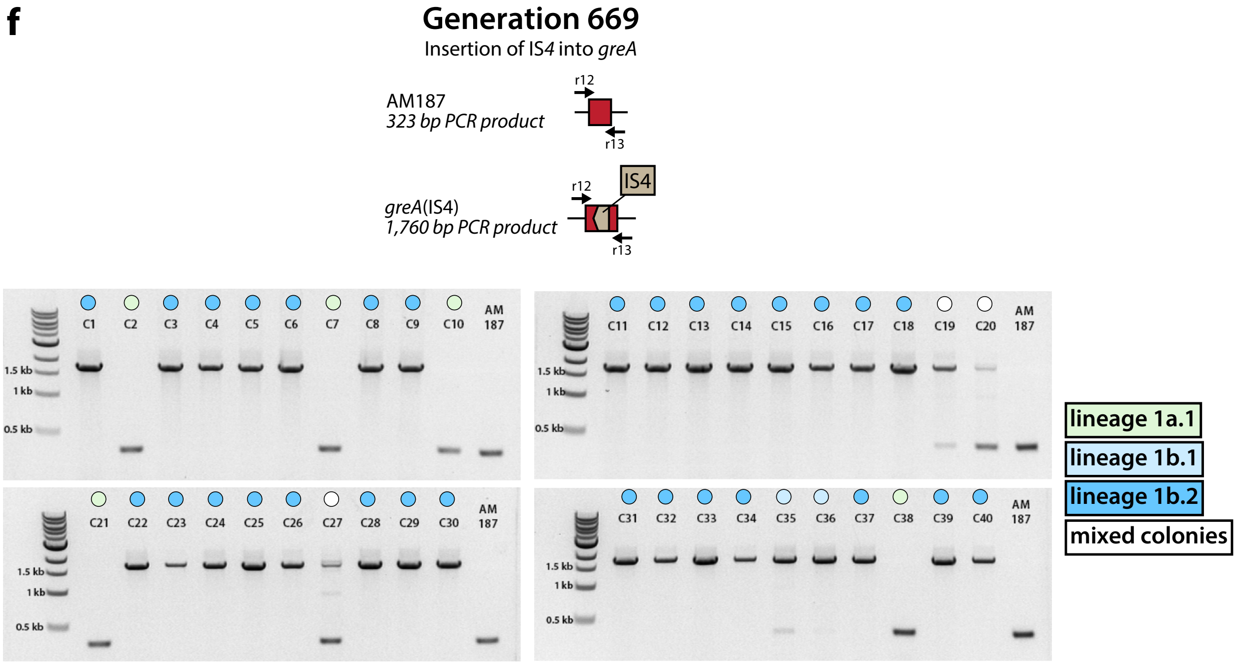

Fig. S5. PCR analysis of the genotypes of forty colonies isolated from population 2 at generation 669 with primers as indicated. Three of the colonies (C19, 20 and 27) showed evidence of the presence of two lineages. **a**, the Clade 1 amplification is present in only 5 of the 37 pure colonies. **b**, Some lineage 1a.1 colonies have only A2 (indicated by the presence of a 1.7 kb band), and others have both A1 and A2 (indicated by the presence of both a 0.5 kb and a 1.7 kb band). **c**, A 110 kb deletion in A2 generated A3 in lineage 1a.1. The 110 kb deletion includes 109 kb of

genomic DNA and 1 kb of IS*5* DNA. **d**, A 132 bp deletion occurred in lineage 1a.1 (indicated by the presence of a 702 bp band). The double bands in colonies 19, 20 and 27 indicate that both lineage 1a.1 and lineage 1b cells were present in the colony. **e**, Evidence for the 4.9 kb amplicon in lineage 1b. **f**, Insertion of IS*4* into *greA* is indicated by the presence of the 1.8 kb band. The double bands in colonies 19, 20 and 27 indicate that both lineage 1a.1 and lineage 1b cells were present in the colony.

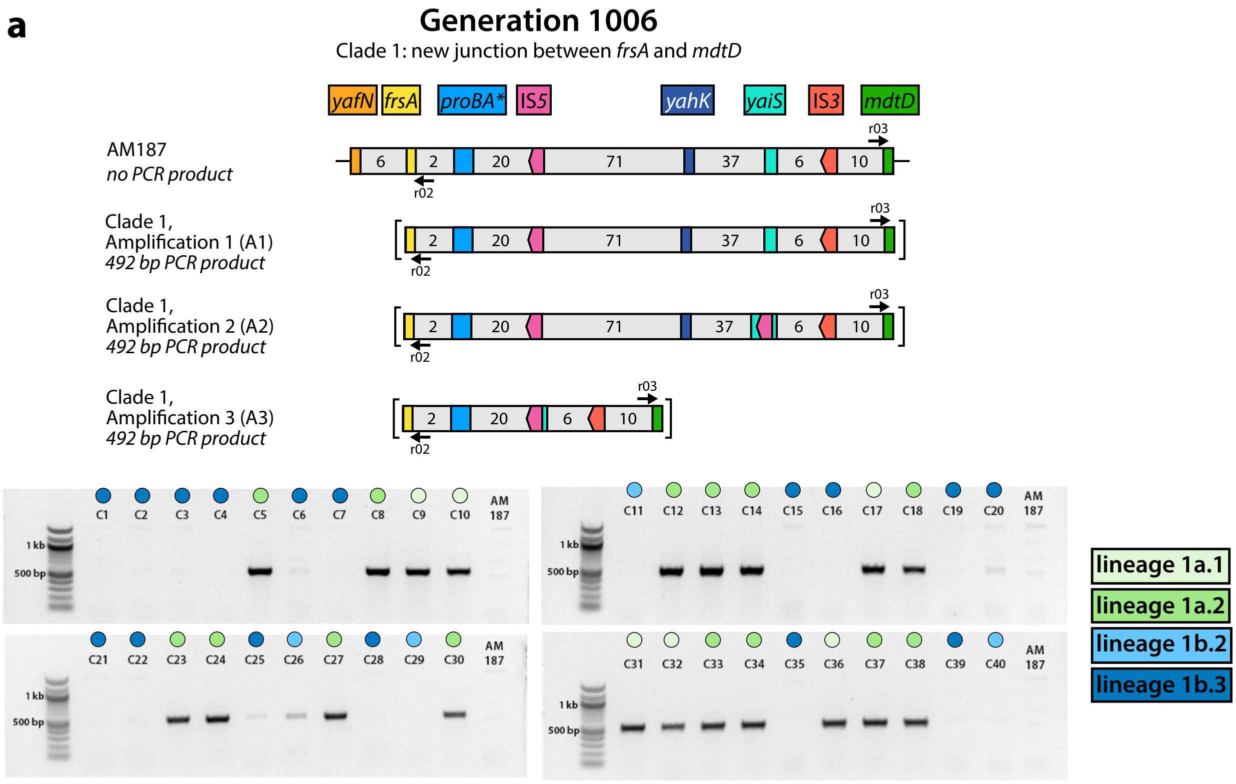
a
Generation 1006
Clade 1: new junction between frsA and mdtD
yafN
frsA
proBA*
IS5
yahK
yaiS
IS3
mdtD
AM187
no PCR product
Clade 1,
Amplification 1 (A1)
492 bp PCR product
Clade 1,
Amplification 2 (A2)
492 bp PCR product
Clade 1,
Amplification 3 (A3)
492 bp PCR product
r02
r03
1 kb
500 bp
AM 187
lineage 1a.1
lineage 1a.2
lineage 1b.2
lineage 1b.3

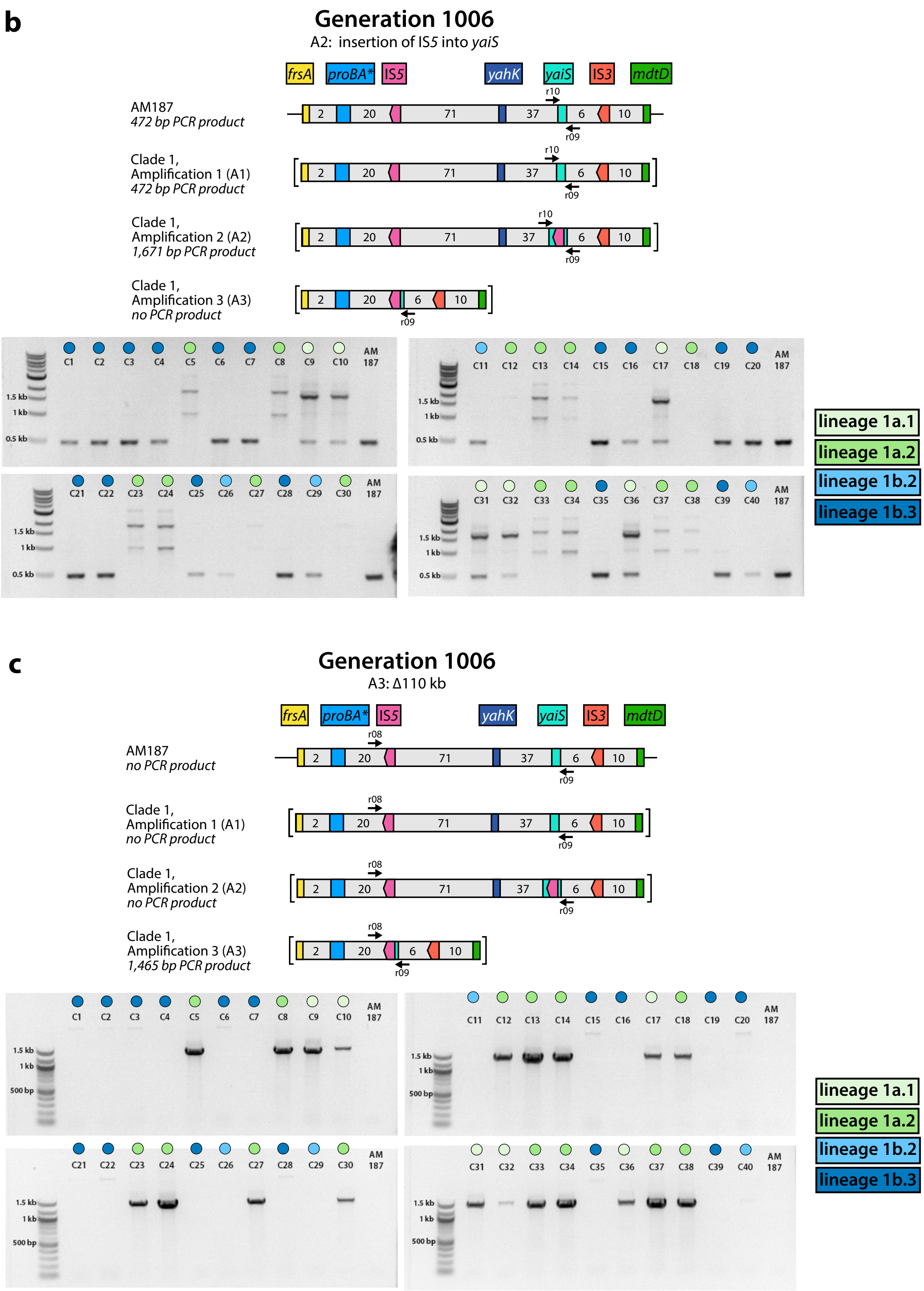
b
Generation 1006
A2: insertion of IS5 into yaiS
frsA
proBA*
IS5
yahK
yaiS
IS3
mdtD
AM187
472 bp PCR product
Clade 1,
Amplification 1 (A1)
472 bp PCR product
Clade 1,
Amplification 2 (A2)
1,671 bp PCR product
Clade 1,
Amplification 3 (A3)
no PCR product
r10
r09
2
20
71
37
6
10
1.5 kb
1 kb
0.5 kb
C1 C2 C3 C4 C5 C6 C7 C8 C9 C10 AM 187
C11 C12 C13 C14 C15 C16 C17 C18 C19 C20 AM 187
C21 C22 C23 C24 C25 C26 C27 C28 C29 C30 AM 187
C31 C32 C33 C34 C35 C36 C37 C38 C39 C40 AM 187
lineage 1a.1
lineage 1a.2
lineage 1b.2
lineage 1b.3
c
Generation 1006
A3: Δ110 kb
frsA
proBA*
IS5
yahK
yaiS
IS3
mdtD
AM187
no PCR product
Clade 1,
Amplification 1 (A1)
no PCR product
Clade 1,
Amplification 2 (A2)
no PCR product
Clade 1,
Amplification 3 (A3)
1,465 bp PCR product
r08
r09
2
20
71
37
6
10
1.5 kb
1 kb
500 bp
C1 C2 C3 C4 C5 C6 C7 C8 C9 C10 AM 187
C11 C12 C13 C14 C15 C16 C17 C18 C19 C20 AM 187
C21 C22 C23 C24 C25 C26 C27 C28 C29 C30 AM 187
C31 C32 C33 C34 C35 C36 C37 C38 C39 C40 AM 187
lineage 1a.1
lineage 1a.2
lineage 1b.2
lineage 1b.3

d

Generation 1006

132 bp deletion in *carB*

Expected band size wt *carB:* 834 bp

Expected band size with Δ132 bp: 702 bp

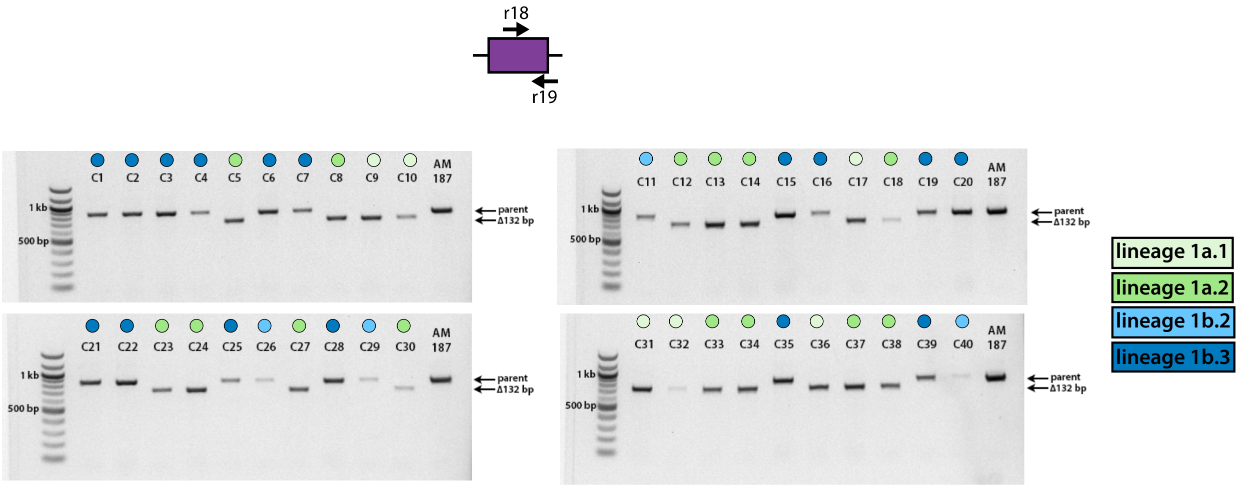

e

Generation 1006

A4: 4.9 kb amplification junction

Expected band size: 537 bp

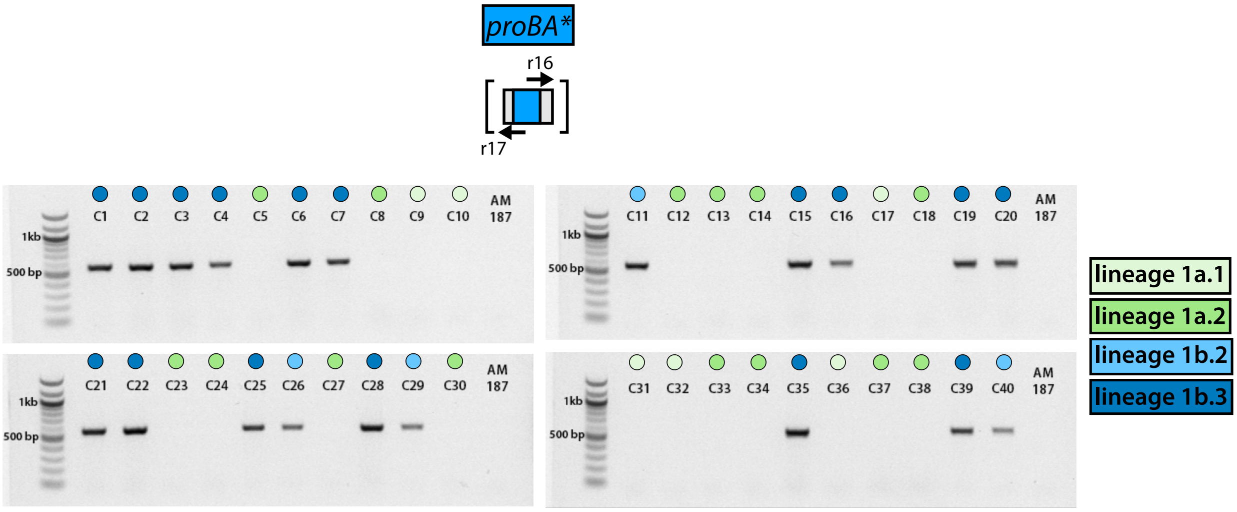

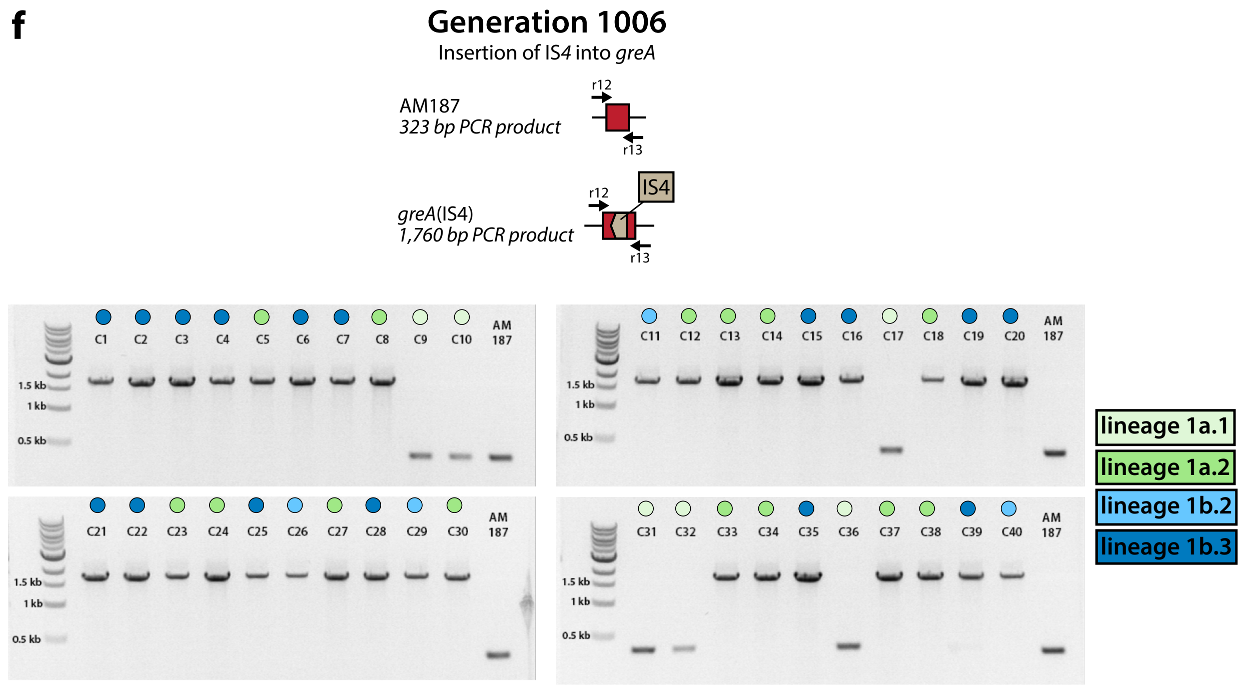

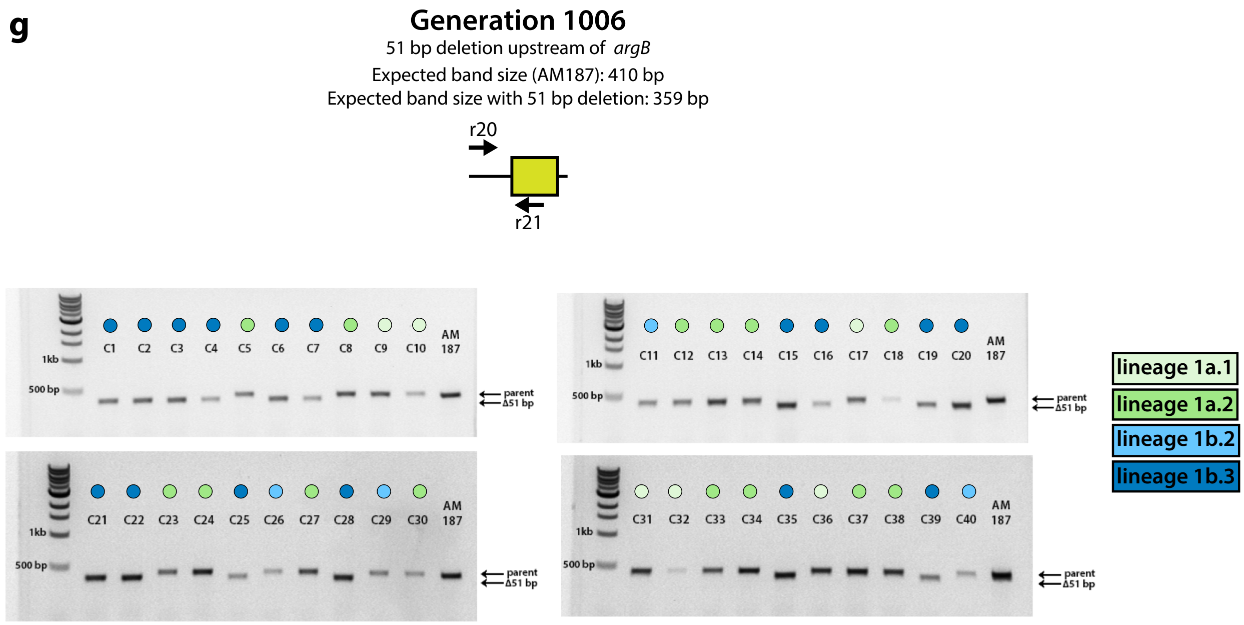

Fig. S6. PCR analysis of the genotypes of forty colonies isolated from population 2 at generation 1006 with primers as indicated. **a**, The Clade 1 amplification junction is present only in lineage 1a.1 and 1a.2, and has been lost in lineage 1b, consistent with reversion to a single copy. **b**, Lineage 1a.1 retains amplicons A1 and A2, as indicated by the 1.7 kb and 0.5 kb bands. The two bands of unexpected sizes visible in some colonies (e.g. C5) are artifacts arising from binding of

primers elsewhere in the genome. The 1 kb band was sequenced and found to map to a region containing *yicO* and *ade.* We were unable to isolate enough of the larger 2 kb band for sequencing. Whole-genome sequencing of two colonies (C5 and C12) did not reveal any rearrangements that would change the sizes of the expected 0.5 and 1 kb bands. **c**, A3 is present in lineages 1a.1 and 1a.2, and is the only amplicon present in lineage 1a.2 (based upon the loss of A1 and A2 (see **b**). **d**, The 132 bp deletion in *carB* is a signature of the 1a lineage. **e**, A4 is found in lineage 1b.2 colonies that no longer have the original Clade 1 amplification junction (see **a**) and have therefore de-amplified A1 to a single copy. **f**, The insertion of IS*4* into *greA* that occurred in lineage 1b by generation 669 also occurred in lineage 1a.2 by generation 1006. **g**, A 51 bp deletion upstream of *argB* occurred in lineage 1b.3.

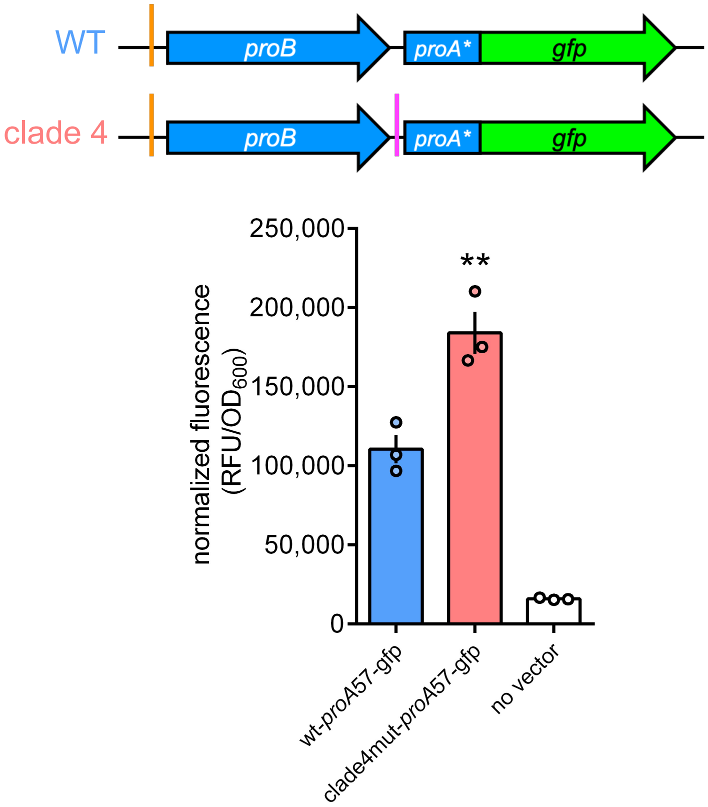

Fig. S7. A G to A mutation at -4 relative to the start codon of *proA** in Clade 4 increases translation efficiency. **a**, The translational fusion used to determine the effect of the mutation at -4. The gold bar indicates the promoter mutation that was introduced into the parental strain AM187 before the evolution of population 2. The magenta bar indicates the G to A mutation at -4 relative to the start codon of *proA**. **b**, The mutation at -4 increases expression of *gfp* by 1.8-fold. **, $p < 0.0025$.

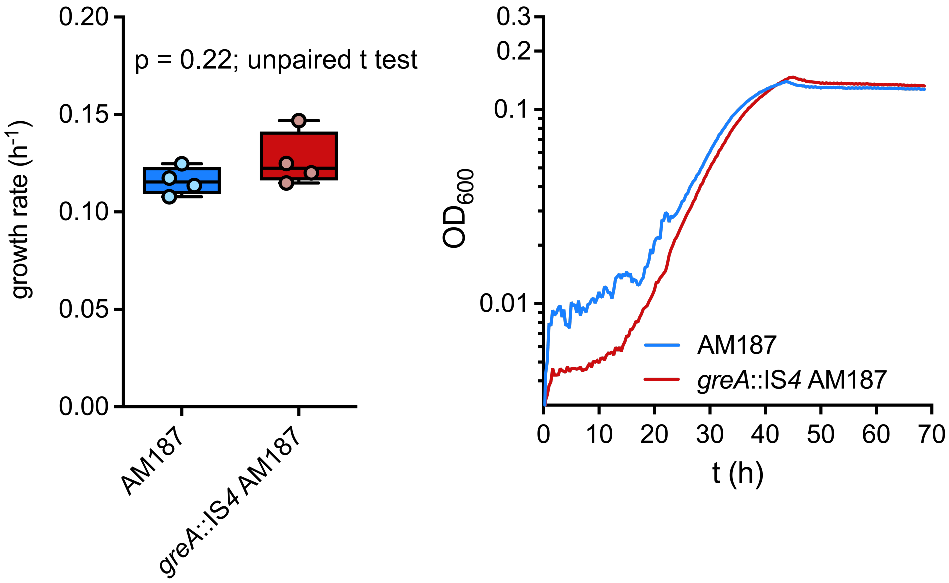

Fig. S8. Insertion of IS*4* into *greA* does not increase growth rate. Cultures were grown in M9 medium containing 0.2% glucose, 0.4 mM proline and 20 µg/mL kanamycin with shaking at 37 ºC.

References

Bossi L, Ratel M, Laurent C, Kerboriou P, Camilli A, Eveno E, Boudvillain M, Figueroa-Bossi N. 2019. NusG prevents transcriptional invasion of H-NS-silenced genes. PLoS Genet 15:e1008425.

Kershner JP, Yu McLoughlin S, Kim J, Morgenthaler A, Ebmeier CC, Old WM, Copley SD. 2016. A synonymous mutation upstream of the gene encoding a weak-link enzyme causes an ultrasensitive response in growth rate. J Bacteriol 198:2853-2863.

Yang DD, Alexander A, Kinnersley M, Cook E, Caudy A, Rosebrock A, Rosenzweig F. 2020. Fitness and productivity increase with ecotypic diversity among *Escherichia coli* strains that coevolved in a simple, constant environment. Appl Environ Microbiol 86.
